# A data-driven framework linking the connectome to spatial gene expression gradients inspired by chemoaffinity theory

**DOI:** 10.1073/pnas.2516572123

**Published:** 2026-03-03

**Authors:** Jigen Koike, Ken Nakae, Riichiro Hira, Yuichiro Yada, Honda Naoki

**Affiliations:** ^a^Laboratory of Data-driven Biology, Graduate School of Integrated Sciences for Life, Hiroshima University, Higashihiroshima 739-8526, Japan; ^b^Laboratory of Data-driven Biology, Nagoya University Graduate School of Medicine, Nagoya 466-8550, Japan; ^c^Digital Twin Lab, Graduate School of Engineering, University of Fukui, Fukui 910-8507, Japan; ^d^The Exploratory Research Center on Life and Living Systems (ExCELLS), National Institutes of Natural Sciences, Okazaki 444-8787, Japan; ^e^Department of Physiology and Cell Biology, Graduate School of Medical and Dental Sciences, Institute of Science Tokyo, Tokyo 113-8519, Japan; ^f^Center for One Medicine Innovative Translational Research (COMIT), Nagoya University, Nagoya 466-8550, Japan

**Keywords:** connectome, transcriptome, chemoaffinity theory, neural wiring, canonical correlation analysis (CCA)

## Abstract

Understanding how brain-wide neural circuits are genetically wired remains a fundamental question in neuroscience. While Sperry’s chemoaffinity theory [Sperry, *Proc. Natl. Acad. Sci. U.S.A.* **50**, 703–710 (1963)] posits that molecular gradients provide positional cues for axonal projections, its application has been largely limited to localized sensory systems. Here, we present SPERRFY (Spatial Positional Encoding for Reconstructing Rules of axonal Fiber connectivitY), a data-driven framework that operationalizes Sperry’s theory at the whole-brain scale. By integrating connectomic data with spatial transcriptomic profiles from the Allen Mouse Brain Atlas, SPERRFY infers latent positional gradients that underlie axonal wiring. Using canonical correlation analysis (CCA), we extract top gradient pairs that align with observed neural connectivity patterns, capturing both global (interregional) and local (intraregional) organizational principles. Connectivity reconstruction based on these gradients shows strong predictive performance, and permutation-based null models confirm the biological relevance of the inferred structures. Furthermore, SPERRFY can screen for candidate genes that may contribute to positional wiring information, providing molecular insight into the developmental logic of brain-wide circuitry. Our results extend Sperry’s foundational theory beyond the sensory domain, offering a unified, data-driven framework for understanding genetically encoded connectivity across the entire brain.

## Significance

Understanding how gene expression patterns determine the wiring of the brain is a fundamental question in neuroscience. SPERRFY offers a data-driven framework to decode spatial encoding of whole-brain connectivity by applying multivariate analysis to connectomic data and spatial gene expression patterns, grounded in Sperry’s chemoaffinity theory. Using mouse brain data, we identified specific gradient patterns that capture major aspects of the connectome’s wiring pattern. These findings place connectome-scale wiring patterns in line with key principles of the chemoaffinity theory, and also provide a versatile tool for mapping genetic determinants of brain-wide neural circuits. By integrating transcriptomics with connectomics, our approach potentially opens avenues for exploring the design principles underlying brain architecture.

The structure of brain circuits is extremely complex and is the basis of higher brain functions such as recognition. One of the most fundamental questions in brain science is how these complex circuits are genetically designed and wired. Many molecular biologists have extensively investigated how neural circuits are wired through axon guidance, in which axons elongate and chemotactically migrate in an attractive or repulsive manner depending on the type of guidance cues (1–3). So far, a large number of guidance cues, e.g., netrin (4, 5), semaphoring (6), ephrin (7–13), and their receptors have been identified. However, these microscopic molecular findings are insufficient for understanding the wiring of the entire brain.

**Fig. 1.**
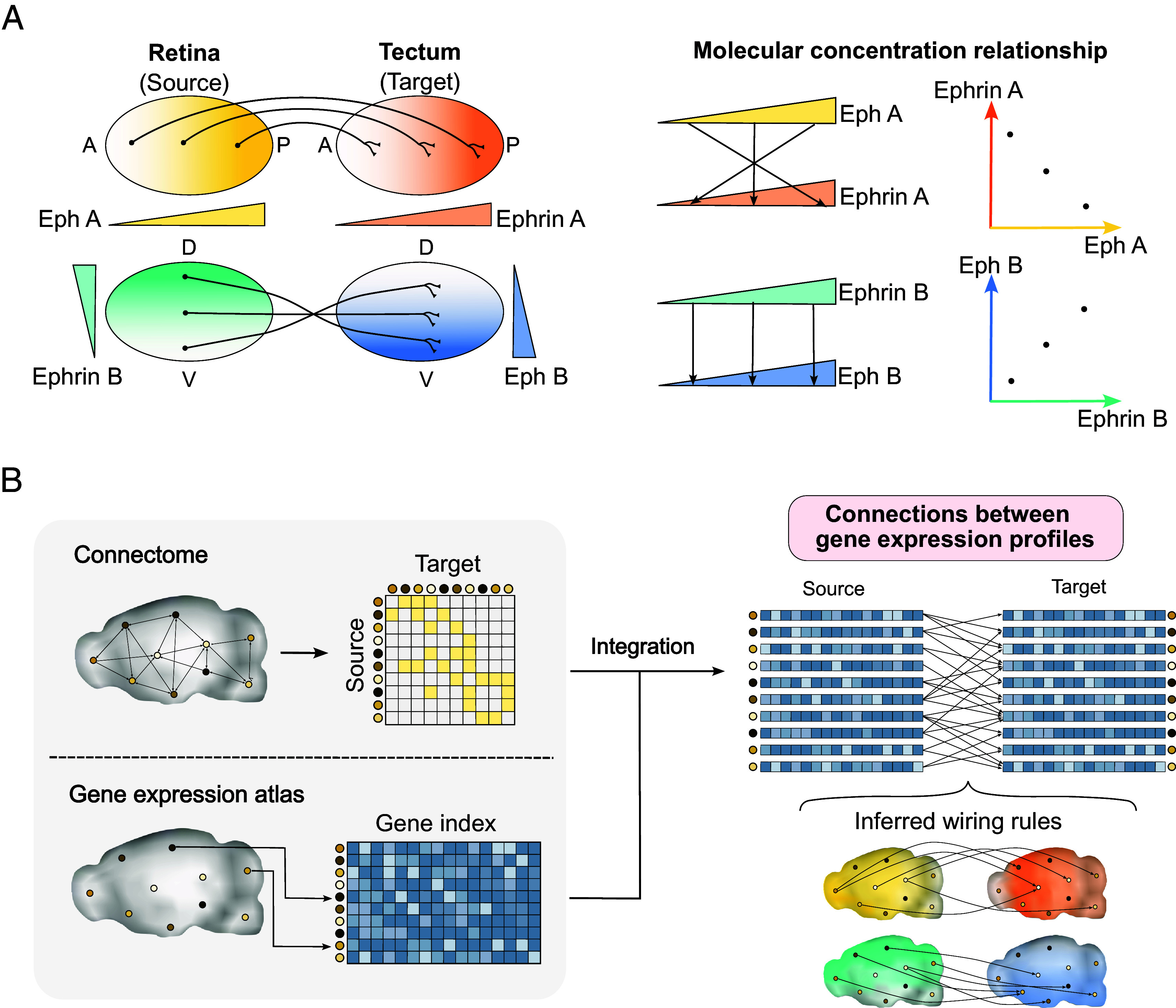
Schematic overview of the chemoaffinity theory and the framework of SPERRFY. (*A*) Conceptual illustration of the chemoaffinity theory in the topographic organization of the visual system. (*Left*) Eph receptors and ephrin ligands are expressed as gradients in the source region (retina) and the target region (tectum), respectively. Axonal projection sites are determined based on the concentration levels of these molecules. Different combinations of molecular gradients along the anterior–posterior (AP) and dorsal–ventral (DV) axes regulate projection specificity. (*Right*) Schematic representation of the molecular concentration relationship between source and target sites. (*B*) Overview of the SPERRFY framework. (*Left*) SPERRFY integrates connectome data and spatial gene expression data (transcriptome atlas). (*Right*) By combining both datasets, latent molecular gradients underlying neural wiring can be inferred, revealing projection rules consistent with the chemoaffinity theory.

In neural circuit formation, neurons must not only project axons chemotactically, but also correctly recognize their projection destination. In 1963, Roger Sperry proposed the chemoaffinity theory, which states that neurons rely on chemical gradients as positional information (PI) to recognize the destination of axon projections (14, 15). Numerous studies supported this chemoaffinity theory and identified a large number of molecules that carry PI (10). For example, in the topographic circuit of the visual system, Eph receptors and ephrin ligands are expressed in gradients in the retina (source region) and the tectum (target region), respectively (16–19) (Fig. 1*A*). The neurons in the source region project their axons to the target region, where they reach the position with a specific ephrin concentration depending on the expression level of Eph receptors in the source region (18, 19). In other words, the concentration gradients of Eph and ephrin are responsible for the “*wiring positional information* (wiring PI)” underlying the genetically programmed neural wiring. However, studies of the chemoaffinity theory have thus far been limited to simple circuits, mainly sensory circuits with topographic structure, e.g., visual and olfactory systems (20, 21). Due to the complexity of brain development, few studies have experimentally investigated complex neural circuits such as those of the cerebrum.

Meanwhile, the structure of the whole brain has been thoroughly mapped in recent years. The brain’s neural wiring pattern (connectome) has been revealed in several animal models, including humans (22, 23), mice (24), marmosets (25, 26), and Drosophila (27–30). In addition, spatial transcriptomic data —the spatial distribution of genome-wide gene expression in the brain— has also been obtained (31–37). These data on connectomes and spatial transcriptomes are now available in public databases. Many data science studies have investigated such data, revealing the relationship between neural connectome patterns and gene expression (38–41). However, despite the availability of such data, no studies have focused on the wiring PI of neural circuits from the perspective of chemoaffinity theory.

In this study, we developed a data-driven framework named “SPERRFY” (Spatial Positional Encoding for Reconstructing Rules of axonal Fiber connectivitY) to decipher wiring PI in actual brain data based on the chemoaffinity theory (Fig. 1*B*). This method employs a machine learning technique called canonical correlation analysis (CCA) (42) to extract hidden relationships between gene expression vectors at the source and target of neural projections. By applying our method to data from the Allen Brain Atlas (24, 32, 33, 43), we extracted pairs of concentration gradients that best explain the actual neural wiring patterns in the mouse brain. These patterns are explained in the context of the chemoaffinity theory as wiring PI. We then verified the validity of the chemoaffinity theory throughout the whole mouse brain, by reconstructing neural wiring patterns using the identified wiring PI. Furthermore, we compared these results to those of null models, suggesting that both local and global control mechanisms underlie the brain’s neural circuitry. Therefore, the SPERRFY framework extends the understanding of the chemoaffinity theory from simple sensory circuits to more complex brain regions.

## Results

### Overview of SPERRFY Framework.

**Fig. 2.**
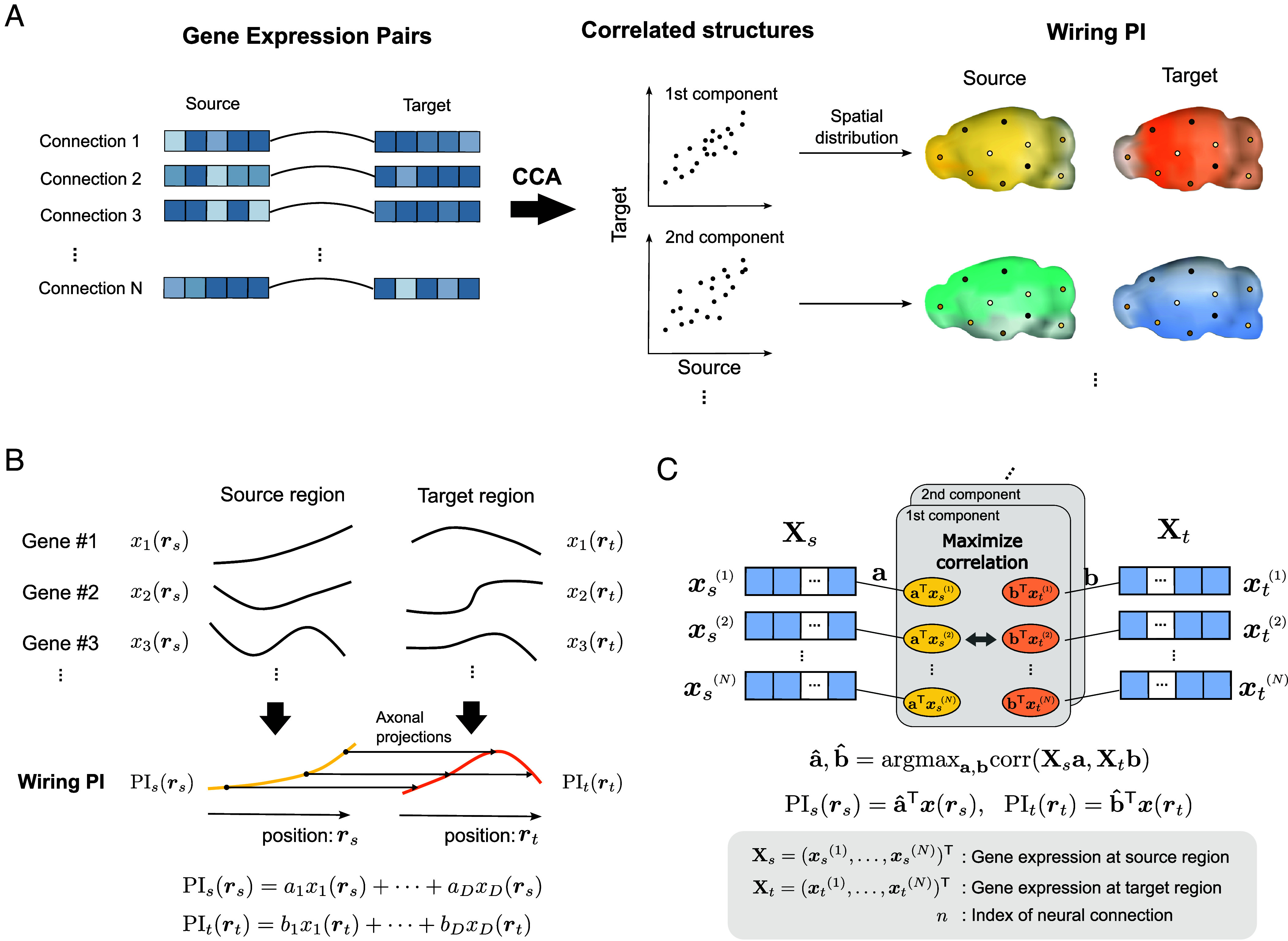
Workflow of SPERRFY and its theoretical basis. (*A*) Workflow of the SPERRFY framework. (*Left*) SPERRFY utilizes integrated connectome and gene expression atlas data, represented as paired gene expression profiles between the source and target sites of neural projections. (*Middle*) By applying canonical correlation analysis (CCA), SPERRFY identifies multiple correlated structures between the source and target sites. (*Right*) The spatial distributions of each wiring PI pair are visualized by mapping their gradients onto the brain regions involved in the connections. (*B*) Formulation of SPERRFY as an extension of the chemoaffinity theory. For simplicity, this model is illustrated in one-dimensional space. In this framework, wiring positional information (PI) is assumed to emerge from the combined influence of multiple gene expression patterns, rather than from individual molecular gradients such as Eph/ephrin. Axonal projections are assumed to form between source and target sites with similar PI values. (*C*) Conceptual diagram of CCA, which identifies pairs of linear combinations from two datasets ($X_{s}$ and $X_{t}$) that maximize their correlation.

“SPERRFY” (Spatial Positional Encoding for Reconstructing Rules of axonal Fiber connectivitY) is designed to discover wiring positional information (wiring PI), that is, latent quantitative gradients that best explain actual neural circuits based on the chemoaffinity theory, by using paired gene expression profiles at source and target sites of neural connections (Fig. 2*A*). According to the chemoaffinity theory, neural wiring is determined by the molecular concentration gradients between source and target sites, resulting in a high correlation between the molecular concentrations at the source and target sites of axonal projections (19).

Leveraging this principle, SPERRFY formulates the *wiring PI* as these weighted combinations of gene-expression levels, thereby providing a quantitative realization of the positional gradients hypothesized in the chemoaffinity theory (Fig. 2*B*). Specifically, the wiring PI at the source (${\text{PI}}_{s}\left(r_{s}\right)$) and target (${\text{PI}}_{t}\left(r_{t}\right)$) sites are defined as

[1] $$\left\{\begin{array}{c} {\text{PI}}_{s}\left(r_{s}\right)=a_{1}x_{1}\left(r_{s}\right)+a_{2}x_{2}\left(r_{s}\right)+\cdots +a_{D}x_{D}\left(r_{s}\right)\\ {\text{PI}}_{t}\left(r_{t}\right)=b_{1}x_{1}\left(r_{t}\right)+b_{2}x_{2}\left(r_{t}\right)+\cdots +b_{D}x_{D}\left(r_{t}\right) \end{array}\right.,$$

where $r_{s}$ and $r_{t}$ represent spatial coordinates of source and target brain region, and $x_{d}\left(r\right)$ denotes the expression levels of the d th gene at position r. SPERRFY assumes that axonal projections preferentially form between regions where ${\text{PI}}_{s}\left(r_{s}\right)$ and ${\text{PI}}_{t}\left(r_{t}\right)$ exhibit similar values. Accordingly, the weight vectors $a={\left(a_{1},\cdots ,a_{D}\right)}^{⊤}$ and $b={\left(b_{1},\cdots ,b_{D}\right)}^{⊤}$ are estimated by CCA, which maximizes correlations between $PI_{s}\left({r_{s}}^{\left(n\right)}\right)$ and $PI_{t}\left({r_{t}}^{\left(n\right)}\right)$ across all observed connections, where n indicates index of the connection.

CCA (42) is an unsupervised machine learning technique for finding correlated structures between paired vector data from different modalities (*SI Appendix*, *Materials and Methods*). In the context of SPERRFY, the two input matrices for CCA are defined as $X_{s}={\left[{x_{s}}^{\left(1\right)},{x_{s}}^{\left(2\right)},\cdots ,{x_{s}}^{\left(N\right)}\right]}^{T}$ and $X_{t}={\left[{x_{t}}^{\left(1\right)},{x_{t}}^{\left(2\right)},\cdots ,{x_{t}}^{\left(N\right)}\right]}^{T}$, where each row vector ${x_{s}}^{\left(n\right)}$ and ${x_{t}}^{\left(n\right)}$ represents the gene-expression vectors of the n-th source (${r_{s}}^{\left(n\right)}$) or target (${r_{t}}^{\left(n\right)}$) region. CCA finds multiple pairs of weight vectors $a^{\left(i\right)}={\left({a_{1}}^{\left(i\right)},\cdots ,{a_{D}}^{\left(i\right)}\right)}^{⊤}$ and $b^{\left(i\right)}={\left({b_{1}}^{\left(i\right)},\cdots ,{b_{D}}^{\left(i\right)}\right)}^{⊤}$ that maximize the correlation between the corresponding weighted projections $X_{s}a^{\left(i\right)}$ and $X_{t}b^{\left(i\right)}$, leading to the corresponding wiring PI gradients $P{I_{s}}^{\left(i\right)}$ and ${\text{PI}}_{t}^{\left(i\right)}$ (Fig. 2*C* and *SI Appendix*, *Materials and Methods*).

The wiring PIs obtained by SPERRFY thus represent the latent molecular gradients that best account for the original connectome data. Furthermore, by reconstructing brain wiring from these positional gradients, we can evaluate the explanatory power of the chemoaffinity theory in actual neural circuits.

### Analysis of Mouse Brain Data From Allen Brain Atlas.

For the application of SPERRFY, we used the Allen Brain Atlas, a public database containing neural connectivity data (24) and gene expression data of adult mice (32, 33). These data can be easily integrated because they have been registered in the same standard brain atlas (43, 44). We transformed these data into paired vector data of gene expression levels to apply the SPERRFY framework (Fig. 2*A*).

**Fig. 3.**
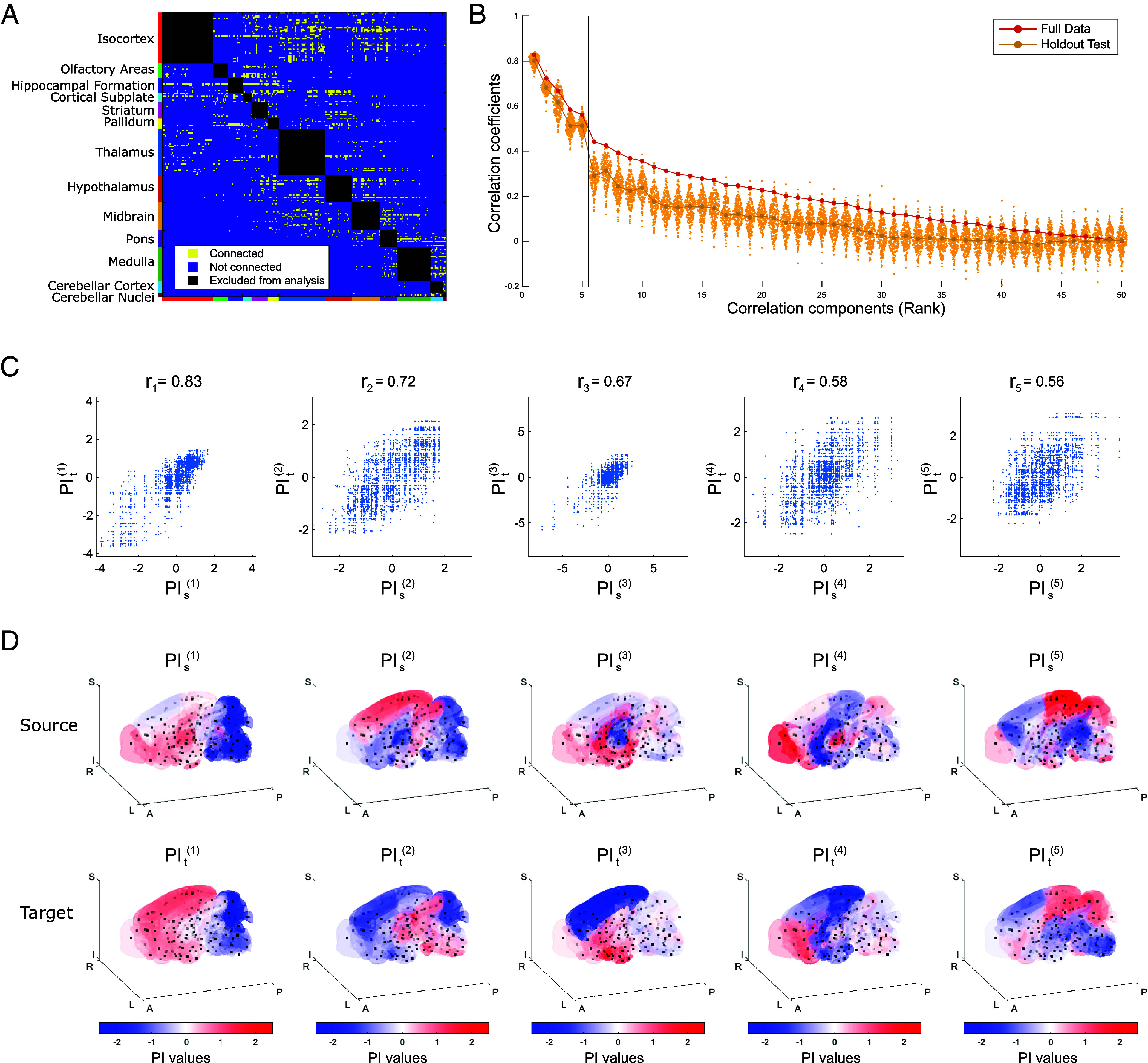
Correlated structures underlying mouse brain wiring extracted by SPERRFY. (*A*) Binary directed matrix representing mesoscale neural connectivity in the mouse brain. Connections within the same major region (MR) are excluded from the analysis. (*B*) Correlation coefficients of the wiring PI components extracted via CCA. Values are ranked in descending order. The red line indicates results from the full dataset, while the yellow line and dots show results from the hold-out validation. (*C*) Scatter plots of the top five most strongly correlated wiring PI components. Each point represents a single neural connection, with x- and y-values corresponding to PI values at the source and target regions. (*D*) Spatial distributions of the top five wiring PI gradient pairs mapped onto the standard mouse brain. Source (*Top* row) and target (*Bottom* row) distributions are shown for each component. Red and blue indicate high and low PI values, respectively.

The neural connectivity data we used are mesoscale connectome data from work by Oh et al. (24), which take the form of a binary matrix representing directed connections between 213 brain regions of the right hemisphere (Fig. 3*A* and *SI Appendix*, *Materials and Methods*). These 213 regions are further divided into 13 major regions (MRs): isocortex, olfactory areas, hippocampal formation, cortical subplate, striatum, pallidum, thalamus, hypothalamus, midbrain, pons, medulla, cerebellar cortex, and cerebellar nuclei (*SI Appendix*, Fig. S1). In our analyses, we focused on connections between different MRs, thereby examining long-range connectivity while excluding short-range connections within the same MR, resulting in a total of 2,213 connections.

The gene expression data are a matrix of the expression levels of 763 genes in these 213 brain regions (*SI Appendix*, Fig. S2A and *Materials and Methods*). In this analysis, gene expression levels were reduced to 50 dimensions using PCA (45) to summarize genes showing redundant spatial expression patterns and to reduce the influence of noise (*SI Appendix*, Fig. S2 *B* and *C*). We integrated this gene expression data and connectivity data into paired vectors of dimensionally reduced gene expression levels in the source and target region of each connection for analysis (*SI Appendix*, *Materials and Methods*).

### Wiring PI of Entire Mouse Brain.

First, we calculated the wiring PI of the entire mouse brain connectivity using data from the Allen Brain Atlas (24, 32, 33, 43). We also performed hold-out validation (46) by randomly dividing the connection matrix into training and test regions, and computing the correlation structures based solely on the training dataset (*SI Appendix*, Fig. S3 and *Materials and Methods*).

By applying SPERRFY, we identified latent correlated structures between the source–target pairs of connections, among which the top 5 exhibited particularly strong correlations (Fig. 3 *B* and *C*). We also confirmed that these top 5 correlated structures were consistently preserved in hold-out validation datasets (Fig. 3*B*). Therefore, the following analysis focused on these top 5 correlated structures.

For these correlated structures, we visualized the identified gradients $P{I_{s}}^{\left(i\right)}\left(r\right)$ and $P{I_{t}}^{\left(i\right)}\left(r\right)$ in the standard mouse brain (44) (Fig. 3*D*). These gradient pairs represent wiring PI and provide the best explanation of the connectome data in the context of the chemoaffinity theory. All five wiring PI gradients were distributed smoothly in the brain with relatively high spatial autocorrelation (47) (*SI Appendix*, Fig. S4*A* and *Materials and Methods*). It was notable that the first PI gradient pair is spatially correlated (*SI Appendix*, Fig. S4*B*) and exhibits a similar spatial pattern, with higher values in the anterior part of the brain and lower values in the posterior part (Fig. 3*D*). The brain’s neural connection matrix exhibits a modular organization, which can be roughly separated into anterior (isocortex, olfactory areas, hippocampal formation, cortical subplate, striatum, pallidum, thalamus, hypothalamus, and midbrain) and posterior (pons, medulla, cerebellar cortex, and cerebellar nuclei) parts (*SI Appendix*, Fig. S5). This most correlated gradient pair may be associated with this modular organization. The second most correlated pair of gradients differs from the most correlated pair, with significantly different distributions between the source and target gradients (Fig. 3*D* and *SI Appendix*, Fig. S4*B*). Notably, some MR pairs—such as isocortex to thalamus, isocortex to midbrain, and midbrain to thalamus—show relatively similar values between source and target, suggesting that this wiring PI may encode preferred directional relationships between specific brain region pairs, rather than reflecting global modularity alone (*SI Appendix*, Fig. S5). The third most correlated pair exhibits a characteristic distribution, with high variance only in the thalamus for the source and the isocortex for the target (*SI Appendix*, Fig. S5). This distribution pattern suggests a potential role in controlling projections from the thalamus to the isocortex. The fourth and fifth gradient pairs exhibit greater variance within each MR compared to the top three correlated pairs. These are more likely to reflect finer-scale connection patterns, i.e., intra-MR connections, rather than inter-MR projection density.

### Genes Showing Similar Distribution to Wiring PI.

**Fig. 4.**
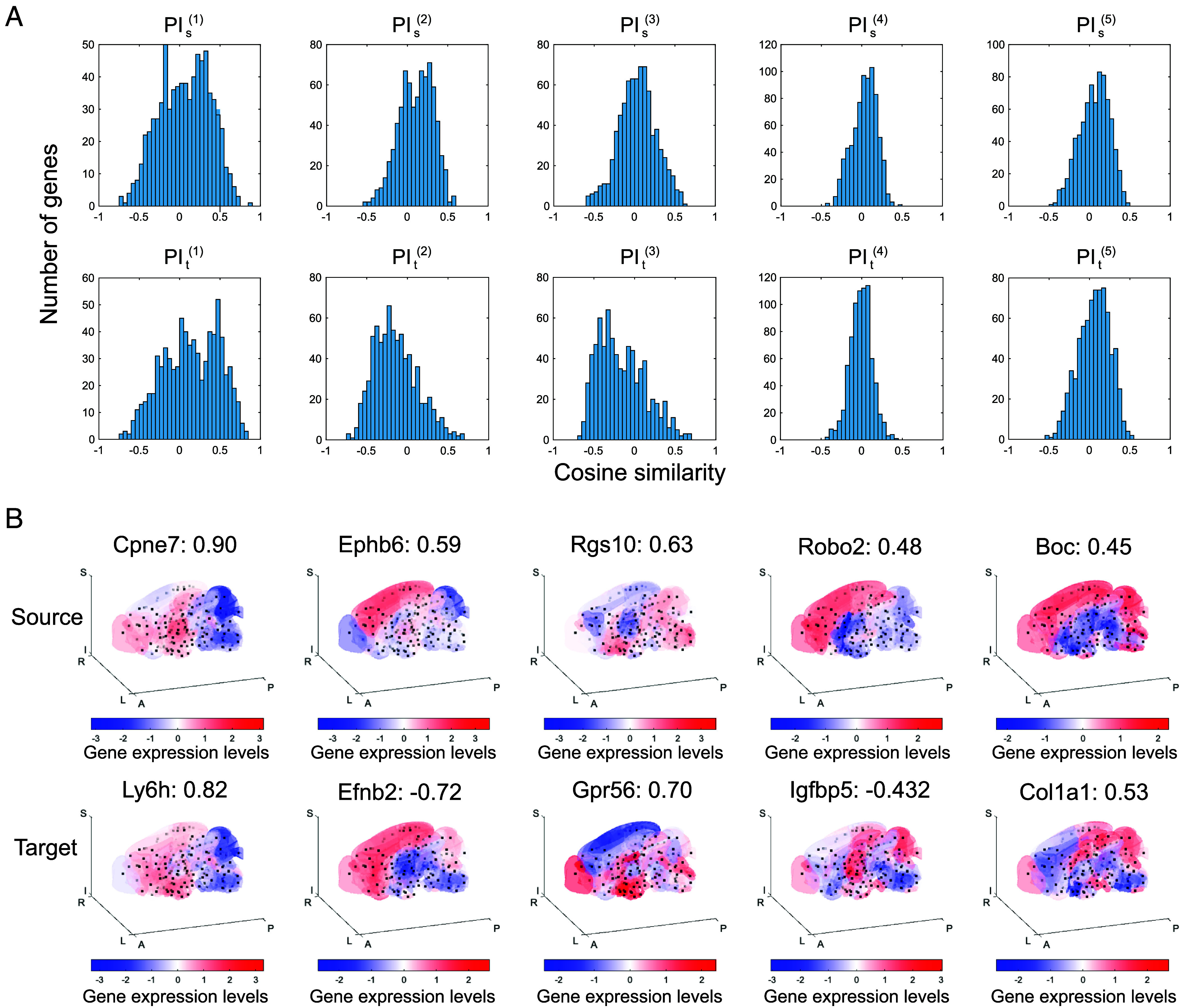
Gene-level correspondence and spatial expression profiles associated with wiring PI gradients. (*A*) Histograms showing the distribution of cosine similarity scores between each wiring PI component and the expression patterns of 763 individual genes. The *Top* row corresponds to source PI components (${\text{PI}}_{s}$), and the *Bottom* row to target PI components (${\text{PI}}_{t}$). (*B*) Spatial gene expression maps of the genes with the highest absolute cosine similarity to each wiring PI gradient. For each component, the most similar gene expression pattern is shown for both source (*Top*) and target (*Bottom*) regions, with the corresponding gene name and similarity score indicated above each map. Red and blue indicate high and low expression levels, respectively.

The inferred wiring PIs could represent spatial molecular gradients underlying axonal wirings. To identify candidate genes associated with the wiring PI pairs, we screened for genes whose spatial expression patterns resemble the identified wiring PI gradients. We calculated cosine similarity scores (*SI Appendix*, *Materials and Methods*) between the spatial expression patterns of 763 genes and each of the extracted wiring PI components (Fig. 4*A*). For each wiring PI, we identified the top-ranked genes and visualized their expression profiles across brain regions (Fig. 4*B*). These genes exhibit distributions similar to the wiring PIs, either in their original form or with inverted signs. The rankings of the top 10 genes with the highest similarity scores for each wiring PI gradient are listed in Table 1. The complete rankings of statistically significant genes are provided in *SI Appendix*, Tables S1 and S2 (see also *SI Appendix*, *Materials and Methods*). For these gene sets, we found no enriched gene ontology (GO) terms (48) (*SI Appendix*, Table S3 and *Materials and Methods*), possibly because the gene set used for analysis is biased toward developmental genes (*SI Appendix*, Fig. S6) and because the significant genes may participate in diverse biological processes. Since the genes showing high correlation with the wiring PI gradients might have been selected merely because of their strong spatial autocorrelation, we tested the relationship between the cosine similarity scores (between wiring PI gradients and spatial gene expression) and the spatial autocorrelation of gene expression, but found no such dependency (*SI Appendix*, Fig. S7).

**Table 1.** Genes with spatial expression patterns most similar to the identified wiring PI gradients

		Source			Target		
PI	Rank	Gene	Similarity	*P*-value	Gene	Similarity	*P*-value
1	1	*Cpne7*	0.90	0.001	*Ly6h*	0.82	0.001
	2	*Nrg1*	−0.74	0.001	*Grm5*	0.81	0.001
	3	*Lhx1*	−0.73	0.006	*Grik5*	0.81	0.001
	4	*Pdgfra*	0.72	0.005	*Cpne7*	0.80	0.001
	5	*Camk2a*	0.72	0.011	*Grin2b*	0.78	0.001
	6	*Ly6h*	0.71	0.003	*Camk2a*	0.78	0.002
	7	*Loxl2*	−0.70	0.001	*Pcdh19*	0.78	0.001
	8	*Pcdh1*	0.69	0.024	*Cacna2d1*	0.77	0.001
	9	*Ctnnb1*	0.68	0.007	*Dlg4*	0.76	0.001
	10	*Grm5*	0.68	0.018	*Lhx1*	−0.75	0.002
2	1	*Ephb6*	0.59	0.001	*Efnb2*	−0.72	0.001
	2	*Adcy2*	0.58	0.001	*Cnr1*	−0.71	0.001
	3	*Chrm3*	0.56	0.001	*Cadps2*	−0.71	0.001
	4	*Cbln2*	0.55	0.002	*Sema3a*	−0.67	0.001
	5	*Lrp1*	0.55	0.001	*Sema6a*	0.67	0.001
	6	*Zic1*	−0.53	0.004	*Amotl1*	0.67	0.001
	7	*Atp8a1*	0.53	0.002	*Tcf7l2*	0.66	0.001
	8	*Arnt2*	0.51	0.002	*Reln*	−0.64	0.001
	9	*Hopx*	−0.50	0.002	*Slc17a6*	0.64	0.002
	10	*L3mbtl1*	0.50	0.003	*Tcf4*	−0.63	0.002
3	1	*Rgs10*	0.63	0.001	*Gpr56*	0.70	0.001
	2	*Parm1*	0.59	0.001	*Nlk*	−0.69	0.001
	3	*Plekhg1*	−0.58	0.001	*Ece2*	0.67	0.001
	4	*Zmat4*	−0.58	0.001	*Baiap3*	0.66	0.001
	5	*Nnat*	0.58	0.001	*Prkcb*	−0.66	0.001
	6	*Kcnj12*	−0.57	0.001	*Dlk1*	0.65	0.001
	7	*Peg10*	0.57	0.001	*Cds1*	−0.64	0.003
	8	*Lef1*	−0.56	0.001	*Zbtb7a*	−0.64	0.001
	9	*Adcyap1r1*	0.56	0.001	*Etv5*	−0.62	0.001
	10	*Hap1*	0.56	0.001	*Slc6a11*	0.62	0.001
4	1	*Robo2*	0.48	0.001	*Igfbp5*	−0.43	0.010
	2	*Slc2a1*	−0.42	0.001	*Tacr1*	0.43	0.004
	3	*C1ql2*	−0.40	0.002	*Nrp1*	−0.40	0.010
	4	*Tshz1*	0.38	0.001	*Pde10a*	0.40	0.015
	5	*Zfp462*	0.35	0.004	*Pbx3*	0.39	0.025
	6	*Mapk8*	0.35	0.007	*Meis1*	0.39	0.053
	7	*Zic1*	−0.33	0.006	*Col15a1*	−0.38	0.030
	8	*Pax8*	0.33	0.007	*Astn2*	−0.37	0.037
	9	*Gsk3a*	0.33	0.002	*Cd9*	−0.37	0.018
	10	*Cux2*	0.32	0.004	*Stx3*	−0.37	0.033
5	1	*Boc*	0.45	0.001	*Col1a1*	0.53	0.001
	2	*Abca2*	−0.45	0.013	*Vat1*	−0.51	0.001
	3	*Mxi1*	0.45	0.008	*Nr4a3*	0.50	0.002
	4	*Nr4a3*	0.45	0.004	*Sncg*	−0.50	0.004
	5	*Ache*	−0.43	0.018	*Zfpm2*	0.50	0.003
	6	*Loxl1*	0.43	0.004	*Cdon*	0.48	0.004
	7	*Neurod1*	0.43	0.019	*Clcn5*	−0.48	0.013
	8	*Slc18a2*	−0.42	0.021	*Adnp2*	0.46	0.019
	9	*Col5a1*	0.42	0.019	*Slc16a2*	0.45	0.011
	10	*Fezf2*	0.42	0.034	*Slc18a2*	−0.44	0.028

The table lists the top 10 genes with the highest cosine similarity to each of the top five wiring PI components, separately for source and target regions. Similarity values were computed across 213 brain regions. A higher absolute value of similarity indicates a stronger correspondence between the gene expression pattern and the wiring PI gradient. *P*-values were calculated by comparing the observed similarity against null distributions generated from spatially constrained surrogate gene expression profiles.

Such genes associated with wiring PI gradients may play an important role in guiding axonal projections during brain development by providing positional molecular cues. For the second PI component, we observed strong similarity to Ephb6 and Efnb2, both members of the Eph/ephrin signaling family known to mediate topographic projections in visual and other sensory systems (10, 14, 18, 19, 49). This correspondence indicates that SPERRFY recovers candidate genes with established links to developmental or wiring-related processes, supporting the biological plausibility of the inferred wiring PI gradients in line with the chemoaffinity framework.

Other PI components also exhibited correspondence with genes potentially relevant to neural wiring. The first PI component showed strong similarity to genes involved in excitatory synaptic transmission, including *Grm5* (mGluR5) (50, 51), *Grin2b* (52), *Dlg4* (PSD-95) (53), and *Camk2a* (54), as well as genes associated with neural development such as *Nrg1* (55) and *Pcdh19* (56). Notably, the source and target PI gradients for this component exhibited highly similar spatial distributions, and several genes—*Camk2a*, *Grm5*, and *Cpne7*—appeared in both rankings. The third PI component showed similarity to *Gpr56* (57, 58), a member of the adhesion-GPCR family known to play essential roles in cortical development and neuronal positioning. The fourth component showed strong similarity to *Robo2* (59), a key axon guidance receptor that mediates Slit signaling and determines axonal projection trajectories during brain development. The fifth component included *Boc* (60, 61), a coreceptor for Sonic Hedgehog (Shh) signaling involved in commissural axon guidance. Together, these results could indicate that the spatial structure of wiring PIs recapitulates the distribution of genes known or suspected to participate in neural circuit formation, suggesting that SPERRFY may capture biologically meaningful PI.

### Reconstructing Connectome Structure Using Wiring PI.

Next, to verify the chemoaffinity theory in the actual whole-brain circuits of mice, we tested whether the identified wiring PI, derived from gene expression data, could be used to reconstruct the neural connectome structure. In the classical chemoaffinity theory, the location of axonal projections is determined by specific molecular concentration gradients (e.g., Eph receptors and ephrin ligands) at the source and target regions. Our SPERRFY framework identifies such concentration gradient pairs as wiring PI from the brain data with the help of CCA. However, it remains uncertain how well the identified wiring PI gradients reflect the actual wiring patterns of the connectome data. To address this issue, we reconstructed neural wiring patterns from identified wiring PIs and compared them to the original connectome matrix.

**Fig. 5.**
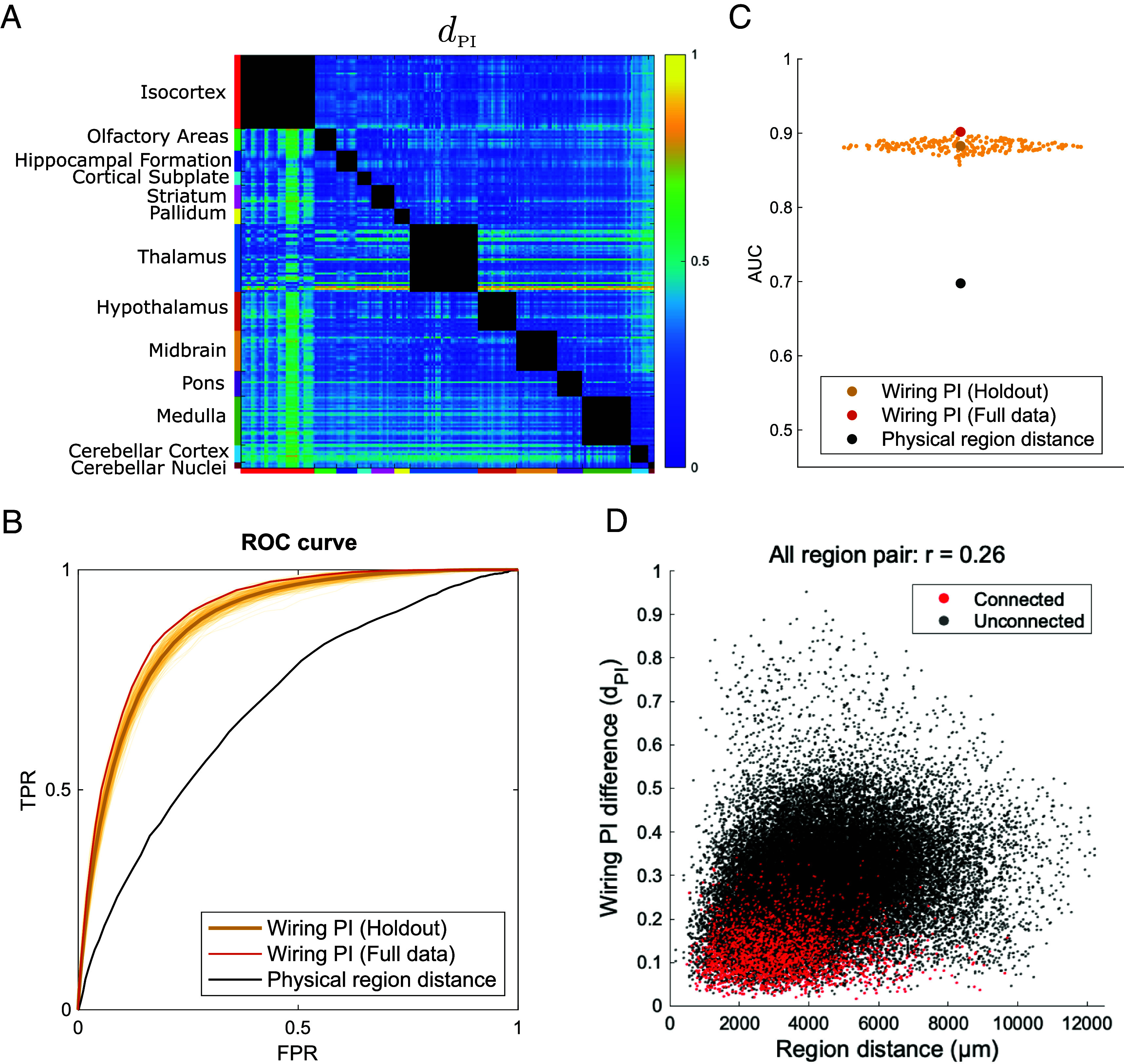
Reconstruction of neural connectivity from wiring PI gradients. (*A*) Matrix used for connection reconstruction, representing the squared Euclidean distance between the top five wiring PI gradients for each region pair. Lower values indicate a higher likelihood of neural connectivity. (*B*) Receiver operating characteristic (ROC) curve for connection reconstruction. The bold yellow line corresponds to the mean ROC across the held-out datasets, and the thin yellow lines correspond to individual held-out datasets (i.e. out-of-sample prediction). The red line corresponds to the results from the full-dataset analysis. The black line corresponds to the reconstruction based on the physical interregional distance matrix. (*C*) AUC values for connection reconstruction. Color coding matches that in panel *B*. (*D*) The relationship between $d_{\mathrm{PI}}\left(r_{s},r_{t}\right)$ and physical interregion distance. Each dot represents a directed (source–target) region pair. Red dots indicate connected pairs (r = 0.03), and black dots indicate unconnected pairs (r = 0.23).

In reconstructing the wiring patterns, we made the following assumption: brain region pairs with similar source and target wiring PI values are more likely to have neural connections, whereas those with dissimilar values are less likely. Based on this assumption, we assessed the extent to which every region pair is likely to be connected from the identified wiring PI gradients. Specifically, we calculated the total difference in the top 5 identified wiring PI gradients as $d_{\mathrm{PI}}\left(r_{s},r_{t}\right)$ (See Eq. **18** in *SI Appendix*, *Materials and Methods*) (Fig. 5*A*). We then reconstructed the binary connection matrix by determining a connection when the value is below the threshold (*SI Appendix*, Fig. S8).

To evaluate the performance of this reconstruction, we generated binary connection matrices with various thresholds and compared them to the original connectome patterns. Then we plotted the receiver operating characteristic (ROC) curves of the connection discrimination by computing the true positive rate (TPR) and false positive rate (FPR). The area under the ROC curve (AUC) on a held-out test set was 0.88, indicating that the reconstructed connectivity patterns closely matched the actual connectome structure (Fig. 5 *B* and *C*). When using the full dataset for both training and evaluation, the AUC remained high at 0.90, suggesting minimal overfitting. We also evaluated whether the wiring PI captures more than just spatial proximity by comparing its predictive power with that of Euclidean distance alone. When replacing $d_{\mathrm{PI}}\left(r_{s},r_{t}\right)$ with the physical interregional distance and applying the same thresholding procedure, the AUC dropped to approximately 0.70, substantially lower than that of the wiring PI-based model. This result demonstrates that wiring PI encodes biologically meaningful information that cannot be explained by geometry alone.

Additionally, we examined the relationship between $d_{\mathrm{PI}}\left(r_{s},r_{t}\right)$ and physical interregional distance (Fig. 5*D*). Across all region pairs, the correlation was weak (r = 0.26). The correlation was nearly absent among anatomically connected pairs (r = 0.03). In contrast, unconnected pairs exhibited a moderately stronger distance dependence (r = 0.23). These findings suggest that wiring PIs encode connectional specificity not trivially accounted for by spatial proximity, further supporting their biological plausibility.

### Comparison of Globally and Locally Randomized Neural Connection Patterns.

**Fig. 6.**
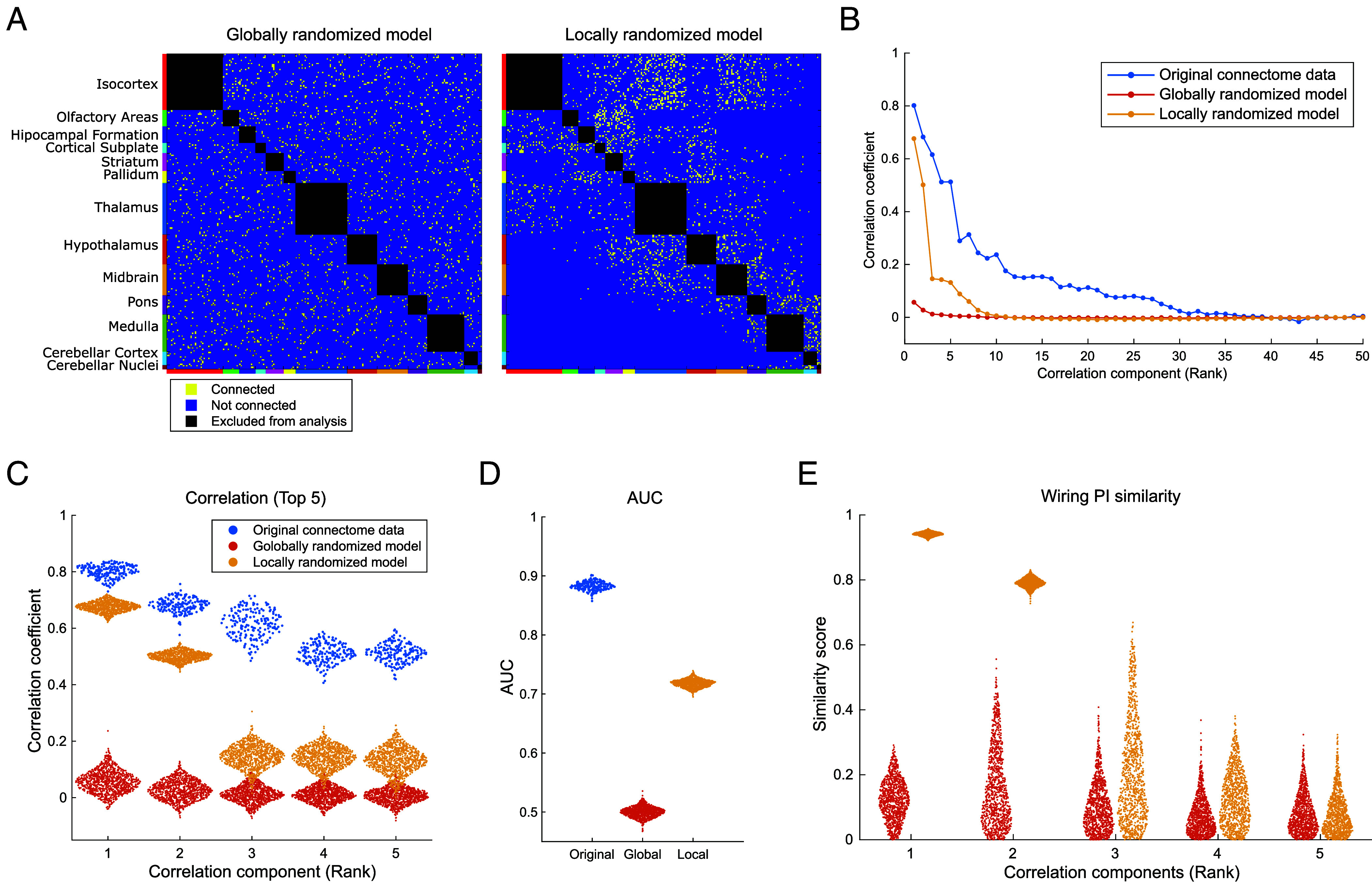
Comparison of SPERRFY results with randomized connection models. (*A*) Example binary connection matrices generated from the globally randomized model (*Left*) and the locally randomized model (*Right*). (*B*) Mean correlation coefficients of SPERRFY-extracted wiring PI components from the original and randomized connectomes. Each value represents the mean across held-out datasets (i.e. out-of-sample prediction). The blue line represents the original data, the red line the globally randomized model, and the yellow line the locally randomized model. (*C*) Distributions of the top five correlation coefficients extracted from each dataset. Color coding matches that in panel *B*. (*D*) Distributions of AUC values for reconstructed connectivity across original and randomized models, evaluated on held-out datasets. (*E*) Similarity scores of wiring PI gradient distributions between randomized and original data.

The above results provide substantial support for the chemoaffinity theory as a model explaining the wiring structure of the mouse brain. Nevertheless, it remains possible that SPERRFY could extract correlated wiring PI gradient structures even from randomly wired connectome data. To evaluate this possibility, we applied SPERRFY to randomized connection patterns (see *SI Appendix*, *Materials and Methods* for details). Specifically, we developed a null model, referred to as the “globally randomized model,” which generates globally randomized connection patterns while preserving the original number of connections (Fig. 6 *A*, *Left*). Using this model, we generated 1,000 randomized connection patterns and applied SPERRFY to each. We confirmed that SPERRFY could not extract correlated wiring PI structures from these random patterns (Fig. 6 *B* and *C*, red line and dots), resulting in extremely low reconstruction performance (red dots in Fig. 6*D*). These findings demonstrate that the SPERRFY framework does not simply extract highly correlated structures by chance, nor does it achieve successful connection reconstruction for the mouse brain connectivity by coincidence.

The globally randomized model served as a useful null model above, but it has limitations in distinguishing global from local effects in neural wiring patterns because it disrupts macroscale connectivity including the brain’s modular organization. We thus developed the “locally randomized model” that disrupts only local connections within each MR pair, while maintaining macroscale connectivity (see *SI Appendix*, *Materials and Methods*). Then we applied SPERRFY to the locally randomized patterns (Fig. 6 *A*, *Right*). The correlation between wiring PI gradients and reconstruction performance were both higher than those of the globally randomized model, but did not achieve the scores of the original data (Fig. 6 *B*–*D*, yellow line and dots). Interestingly, we found that the top two correlated structures robustly exhibited high correlations comparable to those observed in the original connection patterns (Fig. 6*C*). Moreover, the wiring PI gradients of these correlated structures exhibited a similar spatial profile in brain tissue to those obtained from the original mouse brain data (Fig. 6*E* and *SI Appendix*, *Materials and Methods*). These results indicate that the top two wiring PI pairs are not affected so much by local connections within MRs, but reflect global connections between different MR pairs. Because these two gradients show relatively low correlations with each other (*SI Appendix*, Fig. S4*B*), they may function as approximately orthogonal axes defining a coordinate system for large-scale brain organization. In contrast, the third to fifth correlated structures of this null model showed significantly lower correlations than those of the original mouse data (Fig. 6*C*). This means that the third to fifth wiring PIs of the original mouse brain capture information related to local connections, and their correlation structures are disrupted in the locally randomized model. Taken together, these findings suggest the existence of two types of wiring PI—those representing global and local connection patterns—highlighting their potential roles in regulating neural connectivity at multiple scales.

In the above analysis, the globally and locally randomized model were employed to evaluate the wiring PI extracted from the original mouse brain data. However, these null models do not account for key properties of the actual mouse brain, such as connection distances and network topology (62). To evaluate the impact of these factors on the results of SPERRFY, we introduced additional constraints on such properties into the null models (see *SI Appendix*, *Materials and Methods* for details). The results showed that these additional conditions did not significantly alter the trends in correlated structures, although slightly higher correlations and reconstruction performance were observed (*SI Appendix*, Fig. S9). These findings confirm that the wiring PI extracted from the mouse brain data captures substantial and unique information intrinsic to the brain’s actual connectivity.

Finally, we also tested a null model in which the spatial distributions of gene expression were randomized while preserving their spatial autocorrelations (63) (*SI Appendix*, *Materials amd Methods*). When SPERRFY was applied to this spatially randomized dataset, correlated PI gradient structures similar to those obtained from the real data were still extracted, and the reconstruction performance of the connectome was also comparable to that of the original analysis, both showing modest reductions (average AUC = 0.88 in original data, and 0.84 in spatially randomized dataset) (*SI Appendix*, Fig. S10). These results are consistent with the expectation that the wiring PI gradients estimated from the real data can be approximated as weighted combinations of the randomized gene expression patterns. Nevertheless, the higher correlations and reconstruction accuracy observed for the real data indicate that SPERRFY captures biologically meaningful information beyond what can be explained by spatial autocorrelation alone.

## Discussion

In this study, we examined how genetically programmed PI shapes neural wiring across the entire brain by developing the SPERRFY framework as an extension of Sperry’s chemoaffinity theory. By applying SPERRFY to the mouse brain data from the Allen Brain Atlas, we decoded the wiring PI gradients explaining neural wiring patterns according to the chemoaffinity theory. We also successfully reconstructed the original wiring pattern from the spatial profiles of gene expression, thereby supporting the utility of SPERRFY in relating transcriptomic gradients to connectivity in the context of chemoaffinity theory. In addition, we uncovered evidence of hierarchical organization of wiring PI gradients regulating neural wiring at both global and local scales. This hierarchical organization is consistent with the general principles proposed by the Structural Model (64), which describe a hierarchical correspondence between cortical architecture, connectivity, and development. The first few PI gradients exhibit spatial smoothness similar to low-order geometric eigenmodes, consistent with correspondence to large-scale geometric constraints observed in patterns of brain activity (65–67). In this sense, SPERRFY offers a molecular, data-driven complement to the architectonic principles within the cortex. Therefore, SPERRFY provides an analytical framework for evaluating the chemoaffinity theory in actual brain data and for linking molecular gradients to structural hierarchies underlying fundamental wiring principles of the brain.

The proposed SPERRFY framework makes three significant contributions to the field of developmental neuroscience. First, it extends the chemoaffinity theory and applies it to the analysis of complex axonal wiring across multiple brain regions, including higher-order cortical areas. Previous studies have primarily focused on relatively simple and topographic circuits, such as projections from the retina to visual areas (16, 17), peripheral inputs to the somatosensory cortex (68–71), olfactory circuits (72–76), and thalamocortical projections (12, 77–79). In contrast, complex circuits, such as cortico-cortical networks, have rarely been investigated experimentally due to their complexity. In this study, we elucidated wiring principles in the whole brain, including these intricate circuits, that were previously considered beyond the scope of investigation.

Second, SPERRFY provides opportunities to experimentally explore molecules involved in neural wiring. While many molecules, such as Eph and ephrin, have been identified as key regulators of topographic circuits in sensory systems (7–9, 11–13), few molecules have been identified in complex networks such as the cerebral cortex. The SPERRFY framework facilitates the identification of candidate genes responsible for neural wiring by linking the extracted wiring PI to gene expression patterns. Therefore, this study bridges molecular findings with systems-level brain architecture.

Third, the SPERRFY method can be applied to other species and datasets. Although this study focused on the mouse brain, recent advances have developed public databases of neural connectomes and spatial transcriptomes for other species, such as humans (22, 23, 31), Drosophila (27–30, 37), and marmosets (25, 26, 36). Additionally, advancements in measurement technologies are yielding higher-resolution datasets. Applying SPERRFY to such datasets could uncover universal wiring principles across species or reveal species-specific characteristics. The SPERRFY method holds promise as a versatile tool for decoding neural wiring rules across species and developmental stages.

Several computational studies have proposed generative models to explain how genetic, spatial, and structural factors give rise to brain connectivity. In particular, the Genetic Connectome models developed by Barabási and colleagues infer expression-based wiring rules that generate observed connectomic architectures and have yielded testable predictions that can be examined experimentally (80–82). Related frameworks, including those by Oldham et al. (83), Goulas et al. (84), and Song et al. (85), similarly account for network topology through biologically motivated principles such as wiring cost, spatial embedding, and developmental constraints. In contrast to these mechanistic, rule-based approaches, SPERRFY adopts a data-driven statistical perspective, in which correlated gene-expression patterns between source and target regions are identified using CCA to explain the observed connectome. In this sense, SPERRFY provides a chemoaffinity-inspired statistical description of transcriptomic structure underlying brain-wide connectivity and thus occupies a complementary position alongside existing generative models.

In addition, a number of studies have investigated statistical relationships between gene expression and connectivity. For example, French and Pavlidis examined global correspondences between transcriptomic similarity and anatomical connectivity (38), while Fulcher and Fornito identified transcriptional signatures associated with hub-like regions (40). These works established empirical baselines for linking molecular similarity to structural organization. SPERRFY builds upon and extends these descriptive approaches by identifying correlated molecular features between the source and target regions of projections, providing a data-driven yet mechanistically inspired framework for quantitatively examining chemoaffinity-like organization across the whole brain. While SPERRFY does not claim mechanistic causality, its correlation-based formulation captures patterns consistent with the principles originally proposed in the chemoaffinity theory.

Finally, several recent computational inference methods have aimed to predict neural connections directly from molecular or cellular features. For instance, some models have used large-scale gene-expression profiles (86, 87) or cell-type composition data (88) to infer connection probabilities across brain regions. These data-driven methods have been successful in capturing statistical regularities in connectome organization, yet they primarily focus on predictive performance rather than biological interpretability. In contrast, SPERRFY seeks to identify latent molecular axes that are both predictive and interpretable, revealing transcriptomic gradients that may underlie the formation of specific source–target relationships.

The use of brain atlas data necessitates careful consideration of spatial autocorrelation in gene expression to avoid biased results (89). To address this concern, we implemented null models to generate randomized connectome patterns while preserving the actual distribution of neural connection distance. In addition, we implemented other null models designed to account for the network topology of the connectome data because it is also an important characteristic of the actual brain (90, 91). Through statistical testing with these null models, we confirmed significant correlations among the wiring PI gradients extracted by SPERRFY, indicating that the results cannot be fully explained by geometric or topological biases.

Although this research provides deeper insights into neural circuit formation, several limitations should be acknowledged. First, we used data from adult mice and did not address dynamic changes during development. Because chemoaffinity-guided wiring occurs primarily during development, adult transcriptomic gradients may only partially reflect the transient molecular cues that originally guided axon growth. Nevertheless, SPERRFY does not aim to model developmental processes mechanistically but to identify latent molecular gradients that statistically relate to the organization of the existing connectome, interpreting the adult gene-expression landscape as a potential reflection of genetically programmed PI underlying neural wiring during development. This limitation may be mitigated in future studies, as transcriptome data from developmental stages are becoming increasingly available (92–96), although they will need to be matched to adult data for comprehensive analysis. Second, both the transcriptomic and connectomic datasets used in this study have inherent limitations. The gene-expression data from the Allen Brain Atlas are subject to sampling variability and measurement noise, which may blur fine-scale expression gradients. Likewise, the mesoscale connectivity data were binarized to ensure statistical comparability across regions, but this process inevitably discards information about quantitative projection strengths and threshold dependencies. These factors may have influenced the sensitivity of SPERRFY to subtle spatial or molecular differences, highlighting the importance of incorporating more quantitative and higher-resolution datasets in future analyses. Finally, this study primarily focused on correlations and did not establish causal relationships. Future research could address this limitation by experimentally validating the candidate genes identified through SPERRFY. Despite these limitations, this study offers a robust foundation for future investigations into the molecular mechanisms underlying neural circuit formation, facilitating further advances in neuroscience.

## Materials and Methods

We analyzed whole-brain connectome data and spatial gene-expression data obtained from the Allen Brain Atlas. These datasets were analyzed using the SPERRFY framework, which provides a multivariate statistical approach for integrating gene-expression and connectome data. Gene-expression and connectivity data were preprocessed using standard procedures, including normalization and dimensionality reduction. To assess the specificity of the results, multiple null models were constructed and analyzed for comparison. Full technical details of the data preprocessing, analysis pipeline, and validation procedures are provided in the *SI Appendix*, *Materials and Methods*. All code used in the analyses is available on GitHub (https://github.com/JigenKoike/SPERRFY), together with the processed data required to reproduce the results.

## Supplementary Material

Appendix 01 (PDF)

## Data Availability

Source code for analysis data have been deposited in GitHub (https://github.com/JigenKoike/SPERRFY). Study data are included in the article and/or *SI Appendix*. Previously published data were used for this work (24, 32, 33).

## References

[1] M. Tessier-Lavigne, C. S. Goodman, The molecular biology of axon guidance. Science **274**, 1123–1133 (1996). 8895455 10.1126/science.274.5290.1123

[2] B. J. Dickson, Molecular mechanisms of axon guidance. Science **298**, 1959–1964 (2002). 12471249 10.1126/science.1072165

[3] T. W. Yu, C. I. Bargmann, Dynamic regulation of axon guidance. Nat. Neurosci. **4**, 1169–1176 (2001). 10.1038/nn748 11687826

[4] K. Hong, M. Nishiyama, J. Henley, M. Tessier-Lavigne, M. Poo, Calcium signalling in the guidance of nerve growth by Netrin-1. Nature **403**, 93–98 (2000). 10638760 10.1038/47507

[5] M. Nishiyama , Cyclic AMP/GMP-dependent modulation of Ca2+ channels sets the polarity of nerve growth-cone turning. Nature **423**, 990–995 (2003). 12827203 10.1038/nature01751

[6] F. Nakamura, R. G. Kalb, S. M. Strittmatter, Molecular basis of semaphorin-mediated axon guidance. J. Neurobiol. **44**, 219–229 (2000). 10934324 10.1002/1097-4695(200008)44:2<219::aid-neu11>3.0.co;2-w

[7] D. D. M. O’Leary, T. McLaughlin, “Mechanisms of retinotopic map development: Ephs, ephrins, and spontaneous correlated retinal activity” in Progress in Brain Research, Development, Dynamics and Pathiology of Neuronal Networks: from Molecules to Functional Circuits, (Elsevier, 2005), pp. 43–65. 10.1016/S0079-6123(04)47005-8 15581697

[8] G. Scicolone, A. L. Ortalli, N. G. Carri, Key roles of Ephs and ephrins in retinotectal topographic map formation. Brain Res. Bull. **79**, 227–247 (2009). 19480983 10.1016/j.brainresbull.2009.03.008

[9] J. W. Triplett, D. A. Feldheim, Eph and ephrin signaling in the formation of topographic maps. Semin. Cell Dev. Biol. **23**, 7–15 (2012). 22044886 10.1016/j.semcdb.2011.10.026 PMC3288406

[10] J. Egea, R. Klein, Bidirectional Eph–ephrin signaling during axon guidance. Trends Cell Biol. **17**, 230–238 (2007). 17420126 10.1016/j.tcb.2007.03.004

[11] B. Knöll, U. Drescher, Ephrin-As as receptors in topographic projections. Trends Neurosci. **25**, 145–149 (2002). 11852146 10.1016/s0166-2236(00)02093-2

[12] A. Dufour , Area specificity and topography of thalamocortical projections are controlled by ephrin/Eph genes. Neuron **39**, 453–465 (2003). 12895420 10.1016/s0896-6273(03)00440-9

[13] O. Marín, M. J. Blanco, M. A. Nieto, Differential expression of Eph receptors and ephrins correlates with the formation of topographic projections in primary and secondary visual circuits of the embryonic chick forebrain. Dev. Biol. **234**, 289–303 (2001). 11397000 10.1006/dbio.2001.0268

[14] S. L. Zipursky, J. R. Sanes, Chemoaffinity revisited: Dscams, protocadherins, and neural circuit assembly. Cell **143**, 343–353 (2010). 21029858 10.1016/j.cell.2010.10.009

[15] R. W. Sperry, Chemoaffinity in the orderly growth of nerve fiber patterns and connections. Proc. Natl. Acad. Sci. **50**, 703–710 (1963). 14077501 10.1073/pnas.50.4.703 PMC221249

[16] H.-J. Cheng, M. Nakamoto, A. D. Bergemann, J. G. Flanagan, Complementary gradients in expression and binding of ELF-1 and Mek4 in development of the topographic retinotectal projection map. Cell **82**, 371–381 (1995). 7634327 10.1016/0092-8674(95)90426-3

[17] J. Yuasa, S. Hirano, M. Yamagata, M. Noda, Visual projection map specified by topographic expression of transcription factors in the retina. Nature **382**, 632–635 (1996). 8757134 10.1038/382632a0

[18] F. Weth, F. Fiederling, C. Gebhardt, M. Bastmeyer, Chemoaffinity in topographic mapping revisited – Is it more about fiber–fiber than fiber–target interactions? Semin. Cell Dev. Biol. **35**, 126–135 (2014). 25084320 10.1016/j.semcdb.2014.07.010

[19] H. Naoki, Revisiting chemoaffinity theory: Chemotactic implementation of topographic axonal projection. PLoS Comput. Biol. **13**, e1005702 (2017). 28792499 10.1371/journal.pcbi.1005702 PMC5562328

[20] F. Scalia, Synapse formation in the olfactory cortex by regenerating optic axons: Ultrastructural evidence for polyspecific chemoaffinity. J. Comp. Neurol. **263**, 497–513 (1987). 2822778 10.1002/cne.902630404

[21] F. Wang, A. Nemes, M. Mendelsohn, R. Axel, Odorant receptors govern the formation of a precise topographic map. Cell **93**, 47–60 (1998). 9546391 10.1016/s0092-8674(00)81145-9

[22] D. C. Van Essen , The WU-Minn human connectome project: An overview. Neuroimage **80**, 62–79 (2013). 23684880 10.1016/j.neuroimage.2013.05.041 PMC3724347

[23] D. C. Van Essen , The human connectome project: A data acquisition perspective. Neuroimage **62**, 2222–2231 (2012). 22366334 10.1016/j.neuroimage.2012.02.018 PMC3606888

[24] S. W. Oh , A mesoscale connectome of the mouse brain. Nature **508**, 207–214 (2014). 24695228 10.1038/nature13186 PMC5102064

[25] X. Tian , An integrated resource for functional and structural connectivity of the marmoset brain. Nat. Commun. **13**, 7416 (2022). 36456558 10.1038/s41467-022-35197-2 PMC9715556

[26] H. Skibbe , The Brain/MINDS marmoset connectivity resource: An open-access platform for cellular-level tracing and tractography in the primate brain. PLoS Biol. **21**, e3002158 (2023). 37384809 10.1371/journal.pbio.3002158 PMC10337976

[27] L. K. Scheffer , A connectome and analysis of the adult *Drosophila* central brain. eLife **9**, e57443 (2020). 32880371 10.7554/eLife.57443 PMC7546738

[28] S. Dorkenwald , Flywire: Online community for whole-brain connectomics. Nat. Methods **19**, 119–128 (2022). 34949809 10.1038/s41592-021-01330-0 PMC8903166

[29] M. Winding , The connectome of an insect brain. Science **379**, eadd9330 (2023). 36893230 10.1126/science.add9330 PMC7614541

[30] P. Schlegel , Whole-brain annotation and multi-connectome cell typing of *Drosophila*. Nature **634**, 139–152 (2024). 39358521 10.1038/s41586-024-07686-5 PMC11446831

[31] E. H. Shen, C. C. Overly, A. R. Jones, The Allen human brain atlas: Comprehensive gene expression mapping of the human brain. Trends Neurosci. **35**, 711–714 (2012). 23041053 10.1016/j.tins.2012.09.005

[32] E. S. Lein , Genome-wide atlas of gene expression in the adult mouse brain. Nature **445**, 168–176 (2007). 17151600 10.1038/nature05453

[33] L. Ng , An anatomic gene expression atlas of the adult mouse brain. Nat. Neurosci. **12**, 356–362 (2009). 19219037 10.1038/nn.2281

[34] A. M. Henry, J. G. Hohmann, High-resolution gene expression atlases for adult and developing mouse brain and spinal cord. Mamm. Genome **23**, 539–549 (2012). 22832508 10.1007/s00335-012-9406-2

[35] C. L. Thompson , A high-resolution spatiotemporal atlas of gene expression of the developing mouse brain. Neuron **83**, 309–323 (2014). 24952961 10.1016/j.neuron.2014.05.033 PMC4319559

[36] T. Shimogori , Digital gene atlas of neonate common marmoset brain. Neurosci. Res. **128**, 1–13 (2018). 29111135 10.1016/j.neures.2017.10.009

[37] S. W. Robinson, P. Herzyk, J. A. T. Dow, D. P. Leader, Flyatlas: Database of gene expression in the tissues of *Drosophila melanogaster*. Nucleic Acids Res. **41**, D744–D750 (2013). 23203866 10.1093/nar/gks1141 PMC3531048

[38] L. French, P. Pavlidis, Relationships between gene expression and brain wiring in the adult rodent brain. PLoS Comput. Biol. **7**, e1001049 (2011). 21253556 10.1371/journal.pcbi.1001049 PMC3017102

[39] A. Fornito, A. Arnatkevičiūtė, B. D. Fulcher, Bridging the gap between connectome and transcriptome. Trends Cogn. Sci. **23**, 34–50 (2019). 30455082 10.1016/j.tics.2018.10.005

[40] B. D. Fulcher, A. Fornito, A transcriptional signature of hub connectivity in the mouse connectome. Proc. Natl. Acad. Sci. **113**, 1435–1440 (2016). 26772314 10.1073/pnas.1513302113 PMC4747775

[41] B. D. Mills , Correlated gene expression and anatomical communication support synchronized brain activity in the mouse functional connectome. J. Neurosci. **38**, 5774–5787 (2018). 29789379 10.1523/JNEUROSCI.2910-17.2018 PMC6010566

[42] D. R. Hardoon, S. Szedmak, J. Shawe-Taylor, Canonical correlation analysis: An overview with application to learning methods. Neural Comput. **16**, 2639–2664 (2004). 15516276 10.1162/0899766042321814

[43] S. M. Sunkin , Allen brain atlas: An integrated spatio-temporal portal for exploring the central nervous system. Nucleic Acids Res. **41**, D996–D1008 (2013). 23193282 10.1093/nar/gks1042 PMC3531093

[44] Q. Wang , The Allen mouse brain common coordinate framework: A 3D reference atlas. Cell **181**, 936–953.e20 (2020). 32386544 10.1016/j.cell.2020.04.007 PMC8152789

[45] M. Ringnér, What is principal component analysis? Nat. Biotechnol. **26**, 303–304 (2008). 18327243 10.1038/nbt0308-303

[46] S. Yadav, S. Shukla, “Analysis of k-fold cross-validation over hold-out validation on colossal datasets for quality classification” in 2016 IEEE 6th International Conference on Advanced Computing (IACC), (2016), pp. 78–83.

[47] P. a. P. Moran, A test for the serial independence of residuals. Biometrika **37**, 178–181 (1950). 15420264

[48] L. Kolberg , G:Profiler—interoperable web service for functional enrichment analysis and gene identifier mapping (2023 update). Nucleic Acids Res. **51**, W207–W212 (2023). 37144459 10.1093/nar/gkad347 PMC10320099

[49] E. B. Pasquale, Eph-ephrin bidirectional signaling in physiology and disease. Cell **133**, 38–52 (2008). 18394988 10.1016/j.cell.2008.03.011

[50] N. Scheefhals, M. Westra, H. D. MacGillavry, MGLuR5 is transiently confined in perisynaptic nanodomains to shape synaptic function. Nat. Commun. **14**, 1–20 (2023). 36646691 10.1038/s41467-022-35680-w PMC9842668

[51] K. W. Loerwald, A. B. Patel, K. M. Huber, J. R. Gibson, Postsynaptic mGluR5 promotes evoked AMPAR-mediated synaptic transmission onto neocortical layer 2/3 pyramidal neurons during development. J. Neurophysiol. **113**, 786–795 (2015). 25392167 10.1152/jn.00465.2014 PMC4312874

[52] M. Elmasri , Synaptic dysfunction by mutations in GRIN2B: Influence of triheteromeric NMDA receptors on gain-of-function and loss-of-function mutant classification. Brain Sci. **12**, 789 (2022). 35741674 10.3390/brainsci12060789 PMC9221112

[53] A. Rodríguez-Palmero , DLG4-related synaptopathy: A new rare brain disorder. Genet. Med. **23**, 888–899 (2021). 33597769 10.1038/s41436-020-01075-9

[54] J. Lisman, R. Yasuda, S. Raghavachari, Mechanisms of CaMKII action in long-term potentiation. Nat. Rev. Neurosci. **13**, 169–182 (2012). 22334212 10.1038/nrn3192 PMC4050655

[55] O. Chivatakarn, S. Kaneko, Z. He, M. Tessier-Lavigne, R. J. Giger, The Nogo-66 receptor NgR1 is required only for the acute growth cone-collapsing but not the chronic growth-inhibitory actions of myelin inhibitors. J. Neurosci. **27**, 7117–7124 (2007). 17611264 10.1523/JNEUROSCI.1541-07.2007 PMC6794578

[56] A. Pancho , Modifying PCDH19 levels affects cortical interneuron migration. Front. Neurosci. **16**, 887478 (2022). 36389226 10.3389/fnins.2022.887478 PMC9642031

[57] X. Piao , G protein-coupled receptor-dependent development of human frontal cortex. Science **303**, 2033–2036 (2004). 15044805 10.1126/science.1092780

[58] S. Li , GPR56 regulates pial basement membrane integrity and cortical lamination. J. Neurosci. **28**, 5817–5826 (2008). 18509043 10.1523/JNEUROSCI.0853-08.2008 PMC2504715

[59] Y. Gonda, T. Namba, C. Hanashima, Beyond axon guidance: Roles of Slit-Robo signaling in neocortical formation. Front. Cell Dev. Biol. **8**, 607415 (2020). 33425915 10.3389/fcell.2020.607415 PMC7785817

[60] A. Okada , Boc is a receptor for sonic hedgehog in the guidance of commissural axons. Nature **444**, 369–373 (2006). 17086203 10.1038/nature05246

[61] J. Ferent , Boc acts via numb as a Shh-dependent endocytic platform for Ptch1 internalization and Shh-mediated axon guidance. Neuron **102**, 1157–1171.e5 (2019). 31054872 10.1016/j.neuron.2019.04.003

[62] M. Kaiser, A tutorial in connectome analysis: Topological and spatial features of brain networks. Neuroimage **57**, 892–907 (2011). 21605688 10.1016/j.neuroimage.2011.05.025

[63] J. B. Burt, M. Helmer, M. Shinn, A. Anticevic, J. D. Murray, Generative modeling of brain maps with spatial autocorrelation. Neuroimage **220**, 117038 (2020). 32585343 10.1016/j.neuroimage.2020.117038

[64] M. Á. García-Cabezas, B. Zikopoulos, H. Barbas, The structural model: A theory linking connections, plasticity, pathology, development and evolution of the cerebral cortex. Brain Struct. Funct. **224**, 985–1008 (2019). 30739157 10.1007/s00429-019-01841-9 PMC6500485

[65] J. C. Pang , Geometric constraints on human brain function. Nature **618**, 566–574 (2023). 37258669 10.1038/s41586-023-06098-1 PMC10266981

[66] N. C. Gabay, T. Babaie-Janvier, P. A. Robinson, Dynamics of cortical activity eigenmodes including standing, traveling, and rotating waves. Phys. Rev. E **98**, 042413 (2018).

[67] J. A. Henderson, K. M. Aquino, P. A. Robinson, Empirical estimation of the eigenmodes of macroscale cortical dynamics: Reconciling neural field eigenmodes and resting-state networks. Neuroimage Rep. **2**, 100103 (2022). 40567312 10.1016/j.ynirp.2022.100103 PMC12172867

[68] U. C. Drager, D. H. Hubel, Topography of visual and somatosensory projections to mouse superior colliculus. J. Neurophysiol. **39**, 91–101 (1976). 1249606 10.1152/jn.1976.39.1.91

[69] J. H. Kaas, Topographic maps are fundamental to sensory processing. Brain Res. Bull. **44**, 107–112 (1997). 9292198 10.1016/s0361-9230(97)00094-4

[70] M. M. Merzenich , Topographic reorganization of somatosensory cortical areas 3b and 1 in adult monkeys following restricted deafferentation. Neuroscience **8**, 33–55 (1983). 6835522 10.1016/0306-4522(83)90024-6

[71] P. D. Beck, M. W. Pospichal, J. H. Kaas, Topography, architecture, and connections of somatosensory cortex in opossums: Evidence for five somatosensory areas. J. Comp. Neurol. **366**, 109–133 (1996). 8866849 10.1002/(SICI)1096-9861(19960226)366:1<109::AID-CNE8>3.0.CO;2-7

[72] Y. K. Takahashi, M. Kurosaki, S. Hirono, K. Mori, Topographic representation of odorant molecular features in the rat olfactory bulb. J. Neurophysiol. **92**, 2413–2427 (2004). 15152015 10.1152/jn.00236.2004

[73] J. A. Gogos, J. Osborne, A. Nemes, M. Mendelsohn, R. Axel, Genetic ablation and restoration of the olfactory topographic map. Cell **103**, 609–620 (2000). 11106731 10.1016/s0092-8674(00)00164-1

[74] R. Vassar , Topographic organization of sensory projections to the olfactory bulb. Cell **79**, 981–991 (1994). 8001145 10.1016/0092-8674(94)90029-9

[75] M. B. Luskin, J. L. Price, The topographic organization of associational fibers of the olfactory system in the rat, including centrifugal fibers to the olfactory bulb. J. Comp. Neurol. **216**, 264–291 (1983). 6306065 10.1002/cne.902160305

[76] T. Imai, H. Sakano, L. B. Vosshall, Topographic mapping—The olfactory system. Cold Spring Harb. Perspect. Biol. **2**, a001776 (2010). 20554703 10.1101/cshperspect.a001776 PMC2908763

[77] G. López-Bendito, Z. Molnár, Thalamocortical development: How are we going to get there? Nat. Rev. Neurosci. **4**, 276–289 (2003). 12671644 10.1038/nrn1075

[78] S. M. Catalano, R. T. Robertson, H. P. Killackey, Individual axon morphology and thalamocortical topography in developing rat somatosensory cortex. J. Comp. Neurol. **367**, 36–53 (1996). 8867282 10.1002/(SICI)1096-9861(19960325)367:1<36::AID-CNE4>3.0.CO;2-K

[79] Z. Molnár, R. Adams, C. Blakemore, Mechanisms underlying the early establishment of thalamocortical connections in the rat. J. Neurosci. **18**, 5723–5745 (1998). 9671663 10.1523/JNEUROSCI.18-15-05723.1998 PMC6793053

[80] I. A. Kovács, D. L. Barabási, A.-L. Barabási, Uncovering the genetic blueprint of the *C. elegans* nervous system. Proc. Natl. Acad. Sci. **117**, 33570–33577 (2020). 33318182 10.1073/pnas.2009093117 PMC7777131

[81] D. L. Barabási, A.-L. Barabási, A genetic model of the connectome. Neuron **105**, 435–445.e5 (2020). 31806491 10.1016/j.neuron.2019.10.031 PMC7007360

[82] D. L. Barabási, T. Beynon, Á. Katona, N. Perez-Nieves, Complex computation from developmental priors. Nat. Commun. **14**, 2226 (2023). 37076523 10.1038/s41467-023-37980-1 PMC10115783

[83] S. Oldham , Modeling spatial, developmental, physiological, and topological constraints on human brain connectivity. Sci. Adv. **8**, eabm6127 (2022). 35658036 10.1126/sciadv.abm6127 PMC9166341

[84] A. Goulas, R. F. Betzel, C. C. Hilgetag, Spatiotemporal ontogeny of brain wiring. Sci. Adv. **5**, eaav9694 (2019). 31206020 10.1126/sciadv.aav9694 PMC6561744

[85] H. F. Song, H. Kennedy, X.-J. Wang, Spatial embedding of structural similarity in the cerebral cortex. Proc. Natl. Acad. Sci. U.S.A. **111**, 16580–16585 (2014). 25368200 10.1073/pnas.1414153111 PMC4246295

[86] S. Ji, A. Fakhry, H. Deng, Integrative analysis of the connectivity and gene expression atlases in the mouse brain. Neuroimage **84**, 245–253 (2014). 24004696 10.1016/j.neuroimage.2013.08.049

[87] A. Fakhry, S. Ji, High-resolution prediction of mouse brain connectivity using gene expression patterns. Methods **73**, 71–78 (2015). 25109429 10.1016/j.ymeth.2014.07.011

[88] S. Sun, J. Torok, C. Mezias, D. Ma, A. Raj, Spatial cell-type enrichment predicts mouse brain connectivity. Cell Rep. **42**, 113258 (2023). 37858469 10.1016/j.celrep.2023.113258

[89] B. D. Fulcher, A. Arnatkeviciute, A. Fornito, Overcoming false-positive gene-category enrichment in the analysis of spatially resolved transcriptomic brain atlas data. Nat. Commun. **12**, 2669 (2021). 33976144 10.1038/s41467-021-22862-1 PMC8113439

[90] E. Bullmore, O. Sporns, The economy of brain network organization. Nat. Rev. Neurosci. **13**, 336–349 (2012). 22498897 10.1038/nrn3214

[91] W. Gao , Temporal and spatial evolution of brain network topology during the first two years of life. PLoS One **6**, e25278 (2011). 21966479 10.1371/journal.pone.0025278 PMC3179501

[92] H. Li , Mapping fetal brain development based on automated segmentation and 4D brain atlasing. Brain Struct. Funct. **226**, 1961–1972 (2021). 34050792 10.1007/s00429-021-02303-x

[93] E. Braun , Comprehensive cell atlas of the first-trimester developing human brain. Science **382**, eadf1226 (2023). 37824650 10.1126/science.adf1226

[94] F. Jiang , Simultaneous profiling of spatial gene expression and chromatin accessibility during mouse brain development. Nat. Methods **20**, 1048–1057 (2023). 37231265 10.1038/s41592-023-01884-1

[95] M. Sonoda , Six-dimensional dynamic tractography atlas of language connectivity in the developing brain. Brain **144**, 3340–3354 (2021). 34849596 10.1093/brain/awab225 PMC8677551

[96] Y. Li , Spatiotemporal transcriptome atlas reveals the regional specification of the developing human brain. Cell **186**, 5892–5909.e22 (2023). 38091994 10.1016/j.cell.2023.11.016

