# Supplementary material for: A data-driven framework linking the connectome to spatial gene expression gradients inspired by chemoaffinity theory
Source: Proc Natl Acad Sci U S A. 2026 Mar 3;123(10):e2516572123. doi: 10.1073/pnas.2516572123 (PMC12974521; doi:10.1073/pnas.2516572123)
Supplement: Supplementary file 1 — Appendix 01 (PDF) [file pnas.2516572123.sapp.pdf]

PNAS

Supporting Information for
A data-driven framework linking the connectome to spatial gene expression gradients inspired by chemoaffinity theory

Jigen Koike, Ken Nakae, Riichiro Hira, Yuichiro Yada, Honda Naoki

* Honda Naoki
Email: honda.naoki.t1@f.mail.nagoya-u.ac.jp

This PDF file includes:

Supporting text
Figures S1 to S13
Tables S1 to S3

Supplementary Information text

SI Materials & Methods

Mouse connectome data

The mouse connectome data were obtained from the Allen Mouse Brain Connectivity Atlas(24). These connectome data derive from a series of viral microinjection experiments that visualize axonal projections at the mesoscale level and are summarized as a matrix representing connectivity between brain regions. We analyzed the 213 × 213 sub-matrix for the right hemisphere, as listed in Supplementary Table 3 of Oh et al.(24) Following earlier analyses of the same dataset (e.g., Ji et al. 2014(84); Fulcher & Fornito 2016(40)), we binarized the matrix by retaining only those edges with a projection-strength p-value < 0.05, yielding 3,123 directed connections.

The 213 brain regions in the connectivity matrix are grouped into 13 major regions (MRs): isocortex, olfactory areas, hippocampal formation, cortical subplate, striatum, pallidum, thalamus, hypothalamus, midbrain, pons, medulla, cerebellar cortex, and cerebellar nuclei. In our analysis, we focused on inter-MR connectivity, excluding intra-MR connections, in order to minimize the effect of correlated gene expression within the same MR. As a result, the inter-MR connectivity matrix includes 2,213 connections.

Gene expression data of mouse brain

The gene expression data were obtained from the publicly available Allen Mouse Brain Atlas database(32, 33). This database provides summarized results of numerous in situ hybridization (ISH) experiments conducted on the adult mouse brain. Using the Allen API (api.brain-map.org/api/v2/data), we retrieved gene expression datasets corresponding to the same set of 213 regions as used in the connectome data. In summary, 763 genes were selected based on data quality and biological relevance: we first excluded genes with missing values or failed ISH experiments, and then filtered for developmental genes listed in the Allen Developing Mouse Brain Atlas. The details of the data acquisition process are described below.

First, we retrieved identification numbers for all valid ISH experiments (section IDs) by using the following query:

```
api.brain-map.org/api/v2/data/query.json?
criteria=model::SectionDataSet,rma::criteria,
[failed$eq'false'][expression$eq'true'],products[id$eq1]&num_rows=all
```

This query returned 22,157 section datasets containing section IDs. Using these section IDs, gene metadata for each experiment was obtained by querying the following format, replacing "*SectionId*" with the retrieved values:

```
api.brain-map.org/api/v2/data/query.json?
criteria=model::Gene,rma::criteria,data_sets[id$eqSectionId]&num_rows=a
ll
```

To acquire summarized gene expression data for the 213 brain regions, we retrieved structure IDs for all brain structures in the atlas using the following query:

```
api.brain-map.org/api/v2/data/query.json?
criteria=model::Structure,rma::criteria,[graph_id$eq1]&num_rows=all
```

This query yielded 1,327 hierarchical brain structures. We then matched the acronyms in the connectivity matrix to identify the structure IDs corresponding to the 213 brain regions. Finally, gene expression levels for these regions were obtained using the following query format:

```
api.brain-map.org/api/v2/data/query.json?
criteria=model::StructureUnionize,rma::criteria,
structure[id$eqStructureId]&num_rows=all
```

where "StructureId" was replaced with the identified structure IDs. Since the retrieved expression data included unsuccessful experiments, we excluded such data by filtering based on the section IDs of the 22,157 section datasets. Additionally, datasets containing missing values for any of the 213 regions were excluded, resulting in a final set of 9,111 genes. To focus our analysis on

developmental genes, we further restricted our dataset to genes listed in the Allen Developing Mouse Brain Atlas(35), ultimately selecting 763 gene expression datasets.

The datasets of gene expression levels include two types of quantified values: expression density and expression energy. In our analysis, we used expression energy, following previous studies(40, 85). Since ISH measures relative rather than absolute expression levels, we normalized the gene expression data using log transformation and z-scoring. To address redundancy in the distribution of the 763 gene expression datasets, principal component analysis (PCA) was applied. PCA was implemented using the pca function in MATLAB. For the primary analysis, the top 50 principal components (PCs) were selected based on their contribution rates (SI appendix, **Fig. S1**). Additionally, different numbers of PCs (10, 30, 50, 75, and 100) were tested to confirm the effects of the number of PCs (SI appendix, **Fig. S11**).

Integration of connectivity and gene expression data

For the CCA analysis, the connectivity and gene expression data were integrated. Since both datasets shared the same 213 brain regions, this enabled the creation of paired vectors of gene expression levels for all neural connections. Specifically, the binary connectivity data were combined with PCA-reduced gene expression levels from the source and target regions, generating a dataset of paired vectors that served as input for the SPERRFY method. To ensure numerical stability in the CCA computation, a small isotropic Gaussian noise ($\sigma = 0.01$) was added to each of the paired gene expression values, breaking near-linear dependencies without altering the resulting wiring-PI gradients.

Canonical correlation analysis (CCA)

Canonical correlation analysis (CCA) is a machine learning method designed to uncover correlated structures between two sets of variables. Let us consider two multivariate datasets, $\mathbf{X} = (\mathbf{x}_1, \mathbf{x}_2, \dots, \mathbf{x}_N)^\top \in \mathrm{R}^{N\times p}$ and $\mathbf{Y} = (\mathbf{y}_1, \mathbf{y}_2, \dots, \mathbf{y}_N)^\top \in \mathrm{R}^{N\times q}$, where N represents the number of paired observations, and p and q are the numbers of variables (i.e., dimensions) in each dataset. CCA seeks pairs of weighted sums, $u_n = \boldsymbol{a}^\top \mathbf{x}_n$ and $v_n = \boldsymbol{b}^\top \mathbf{y}_n$, such that the correlation between $\boldsymbol{u} = (u_1, u_2, \dots, u_N)^\top \in \mathrm{R}^{N\times 1}$ and $\boldsymbol{v} = (v_1, v_2, \dots, v_N)^\top \in \mathrm{R}^{N\times 1}$ is maximized. Here, $\boldsymbol{a} \in \mathrm{R}^{p\times 1}$ and $\boldsymbol{b} \in \mathrm{R}^{q\times 1}$ are weight vectors optimized by CCA. In other words, CCA addresses the following optimization problem:

$$\widehat{\boldsymbol{a}}, \widehat{\boldsymbol{b}} = \operatorname{argmax}_{\boldsymbol{a},\boldsymbol{b}} \operatorname{corr}(\mathbf{X}\boldsymbol{a}, \mathbf{Y}\boldsymbol{b}) \tag{2}$$

CCA provides not just a single pair of solutions but multiple pairs of $\widehat{\boldsymbol{a}}^{(i)} \in \mathrm{R}^{p\times 1}$and $\widehat{\boldsymbol{b}}^{(i)} \in \mathrm{R}^{q\times 1}$, each associated with a correlated pair of $\boldsymbol{u}^{(i)} = \mathbf{X}\widehat{\boldsymbol{a}}^{(i)}$ and $\boldsymbol{v}^{(i)} = \mathbf{Y}\widehat{\boldsymbol{b}}^{(i)}$. These weight vector pairs are mutually orthogonal (i.e., ${\widehat{\boldsymbol{a}}^{(i)}}^\top \widehat{\boldsymbol{a}}^{(j)} = 0 \ (i \neq j)$), allowing independent patterns of correlation to be extracted. The index (i) is ordered according to the magnitude of the corresponding correlation coefficients.

The derivation of the CCA solution is described as follows. For simplicity, $\mathbf{X}$ and $\mathbf{Y}$ are assumed to be mean-centered. The correlation coefficient $\rho(\boldsymbol{a}, \boldsymbol{b})$, which is maximized in CCA, is expressed as:

$$\rho(\boldsymbol{a}, \boldsymbol{b}) = \frac{\frac{1}{N}\boldsymbol{u}^\top \boldsymbol{v}}{\sqrt{\frac{1}{N}\boldsymbol{u}^\top \boldsymbol{u}}\sqrt{\frac{1}{N}\boldsymbol{v}^\top \boldsymbol{v}}} = \frac{\boldsymbol{a}^\top \mathbf{V}_{\mathrm{XY}} \boldsymbol{b}}{\sqrt{\boldsymbol{a}^\top \mathbf{V}_{\mathrm{XX}} \boldsymbol{a}}\sqrt{\boldsymbol{b}^\top \mathbf{V}_{\mathrm{YY}} \boldsymbol{b}}} \tag{3}$$

where $\mathbf{V}_{\mathrm{XX}} = \frac{1}{N}\mathbf{X}^\top\mathbf{X}$, $\mathbf{V}_{\mathrm{YY}} = \frac{1}{N}\mathbf{Y}^\top\mathbf{Y}$, and $\mathbf{V}_{\mathrm{XY}} = \frac{1}{N}\mathbf{X}^\top\mathbf{Y}$ represent the sample variance-covariance matrices of $\mathbf{X}$ and $\mathbf{Y}$, respectively. In CCA, the objective is to find a pair $(\boldsymbol{a}, \boldsymbol{b})$ that maximizes $\rho(\boldsymbol{a}, \boldsymbol{b})$.

Since multiplying $\boldsymbol{a}$ and $\boldsymbol{b}$ by positive constants does not change $\rho(\boldsymbol{a}, \boldsymbol{b})$, the standard deviations in the denominator can be normalized to 1. Thus, the optimization problem can be rewritten as:

$$\operatorname{argmax}_{\boldsymbol{a},\boldsymbol{b}}\ \boldsymbol{a}^\top \mathbf{V}_{\mathrm{XY}} \boldsymbol{b} \quad \mathrm{s.t.}\ \ \boldsymbol{a}^\top \mathbf{V}_{\mathrm{XX}} \boldsymbol{a} = \boldsymbol{b}^\top \mathbf{V}_{\mathrm{YY}} \boldsymbol{b} = \mathbf{1} \tag{4}$$

This constrained optimization problem can be reformulated using the method of Lagrange's multipliers as follows:

$$\operatorname{argmax}_{\boldsymbol{a},\boldsymbol{b},\lambda_{\boldsymbol{a}},\lambda_{\boldsymbol{b}}} L(\boldsymbol{a}, \boldsymbol{b}, \lambda_{\boldsymbol{a}}, \lambda_{\boldsymbol{b}}) = \boldsymbol{a}^\top \mathbf{V}_{\mathrm{XY}} \boldsymbol{b} + \lambda_{\boldsymbol{a}}(1 - \boldsymbol{a}^\top \mathbf{V}_{\mathrm{XX}} \boldsymbol{a}) + \lambda_{\boldsymbol{b}}(1 - \boldsymbol{b}^\top \mathbf{V}_{\mathrm{YY}} \boldsymbol{b}) \tag{5}$$

Taking the partial derivatives of $L(\boldsymbol{a}, \boldsymbol{b}, \lambda_a, \lambda_b)$ with respect to a and b and setting them to zero yields:

$$\mathbf{V_{XY}}\boldsymbol{b} = 2\lambda_a \mathbf{V_{XX}}\boldsymbol{a} \tag{6}$$

$$\mathbf{V_{XY}}^{\top}\boldsymbol{a} = 2\lambda_b \mathbf{V_{YY}}\boldsymbol{b} \tag{7}$$

By multiplying both sides of the equations by $\boldsymbol{a}^{\top}$ and $\boldsymbol{b}^{\top}$, respectively, we obtain the following relationship:

$$\boldsymbol{a}^{\top}\mathbf{V_{XY}}\boldsymbol{b} = 2\lambda_a = 2\lambda_b = \lambda \tag{8}$$

Thus, the above equations can be expressed together in matrix form as:

$$\begin{pmatrix} O & \mathbf{V_{XY}} \\ \mathbf{V_{XY}}^{\top} & O \end{pmatrix}\begin{pmatrix} \boldsymbol{a} \\ \boldsymbol{b} \end{pmatrix} = \begin{pmatrix} \mathbf{V_{XX}} & O \\ O & \mathbf{V_{YY}} \end{pmatrix}\begin{pmatrix} \boldsymbol{a} \\ \boldsymbol{b} \end{pmatrix} \tag{9}$$

This is a generalized eigenvalue problem, where the eigenvalue λ corresponds to the correlation coefficient $\boldsymbol{a}^{\top}\mathbf{V_{XY}}\boldsymbol{b}$. By focusing on the second and subsequent eigenvalues, up to min$\{p, q\}$ number of correlated components can be obtained.

Application of CCA in the SPERRFY framework

In the framework of SPERRFY, CCA is employed to identify latent correlations between gene expression profiles at the source and target regions of neural connections. Given the paired gene expression data from the source and target regions, CCA extracts linear combinations that maximize their correlation, revealing underlying patterns of wiring positional information (PI).

Let $\boldsymbol{r}_s{}^{(n)}$ and $\boldsymbol{r}_t{}^{(n)}$ represent the spatial coordinate indices of the source and target regions for the nth neural connection, respectively. The gene expression levels at these coordinate indices are denoted as $\boldsymbol{x}(\boldsymbol{r}_s{}^{(n)}) \in \mathrm{R}^{D\times 1}$ and $\boldsymbol{x}(\boldsymbol{r}_t{}^{(n)}) \in \mathrm{R}^{D\times 1}$, where D is the number of dimensions after dimensionality reduction. In SPERRFY, the wiring PI is represented as pairs of weighted sums of gene expression levels at the source and target regions:

$$\mathrm{PI}_s(\boldsymbol{r}_s{}^{(n)}) = \sum_{d=1}^{D} a_d x_d(\boldsymbol{r}_s{}^{(n)}) = \boldsymbol{a}^{\top}\boldsymbol{x}(\boldsymbol{r}_s{}^{(n)}) \tag{10}$$

$$\mathrm{PI}_t(\boldsymbol{r}_t{}^{(n)}) = \sum_{d=1}^{D} b_d x_d(\boldsymbol{r}_t{}^{(n)}) = \boldsymbol{b}^{\top}\boldsymbol{x}(\boldsymbol{r}_t{}^{(n)}) \tag{11}$$

CCA estimates the weight vectors $\boldsymbol{a} \in \mathrm{R}^{D\times 1}$ and $\boldsymbol{b} \in \mathrm{R}^{D\times 1}$ such that the correlation between the wiring PI at the source and target regions is maximized as:

$$\hat{\boldsymbol{a}}, \hat{\boldsymbol{b}} = \mathrm{argmax}_{\boldsymbol{a},\boldsymbol{b}}\ \mathrm{corr}\left(\mathrm{PI}_s(\boldsymbol{r}_s{}^{(n)}), \mathrm{PI}_t(\boldsymbol{r}_t{}^{(n)})\right) \quad (n = 1, \ldots, N) \tag{12}$$

The CCA framework yields multiple pairs of weight vectors $(\hat{\boldsymbol{a}}^{(i)}, \hat{\boldsymbol{b}}^{(i)})$, each corresponding to the ith highest correlation. With these weights, multiple PI gradients across the brain can be computed as:

$$\mathrm{PI}_s{}^{(i)}(\boldsymbol{r}) = \hat{\boldsymbol{a}}^{(i)\top}\boldsymbol{x}(\boldsymbol{r}) \tag{13}$$

$$\mathrm{PI}_t{}^{(i)}(\boldsymbol{r}) = \hat{\boldsymbol{b}}^{(i)\top}\boldsymbol{x}(\boldsymbol{r}) \tag{14}$$

where $\boldsymbol{x}$ indicates a spatial coordinate of the brain. CCA was implemented using the `canoncorr` function in MATLAB.

Hold-out validation with train-test split dataset

To assess the reliability of the wiring PI components extracted by SPERRFY, we conducted hold-out validation (**SI appendix, Fig. S3**). For this validation, the entire neural connectivity matrix was randomly split into a training set (80%) and a test set (20%). In the training phase, CCA was applied exclusively to the paired gene expression data corresponding to the neural connections in the training set, excluding connections in the test set. This ensured that the extracted PI gradients were derived solely from the training data, without contamination from the test set connections. The extracted wiring PI gradients from the training set were then evaluated on the test set by calculating the correlation coefficients between the PI values at the source and target regions of the test set connections (**SI appendix, Fig. S3**). Hold-out validation was also conducted to evaluate the reconstruction of neural wiring from the estimated wiring PI gradients (**Fig. 5B, C**). This analysis

provided a quantitative measure of how well the identified PI gradients capture positional relationship in neural connectivity patterns within unseen data.

Spatial autocorrelation of wiring PI gradients and gene expression patterns

To quantify the spatial autocorrelation of wiring PI gradients and region-wise gene expression patterns, we computed Moran's I(47), a widely used measure of spatial autocorrelation. For a given spatial map $\boldsymbol{x} = (x_1, x_2, \ldots, x_N)$, Moran's I was defined as:

$$I = \frac{N}{S_0} \frac{\sum_{i=1}^{N} \sum_{j=1}^{N} w_{ij} (x_i - \bar{x})(x_j - \bar{x})}{\sum_{i=1}^{N} (x_i - \bar{x})^2} \tag{15}$$

where $\bar{x} = \frac{1}{N} \sum_{i=1}^{N} x_i$ and $S_0 = \sum_{i=1}^{N} \sum_{j=1}^{N} w_{ij}$. Here, w_{ij} denotes the spatial weight between regions i and j. Spatial weights w_{ij} were defined using an exponential decay of inter-regional distance d_{ij}:

$$w_{ij} = exp(-d_{ij}/h) \tag{16}$$

such that nearby regions contribute more strongly to the statistic.
The decay parameter h determines the effective spatial scale of weighting. We screened multiple h values (log-spaced around the median inter-regional distance), and observed that smaller h tended to increase Moran's I for many spatial maps, implying that short-range similarity contributes strongly to spatial autocorrelation. However, very small h values resulted in numerical instability due to a reduced S_0. To balance sensitivity and stability, we selected the smallest h that enabled stable computation.

Similarity between gene expression patterns and wiring PI gradients

To screen genes associated with wiring PI, we evaluated the similarity between gene expression patterns and wiring PI gradients using cosine similarity. Given two D-dimensional vectors $\boldsymbol{x}$ and $\boldsymbol{y}$, cosine similarity $S_c(\boldsymbol{x}, \boldsymbol{y})$ is defined as follows:

$$S_c(\boldsymbol{x}, \boldsymbol{y}) = \frac{\sum_{d=1}^{D} x_d y_d}{\sqrt{\sum_{d=1}^{D} {x_d}^2} \sqrt{\sum_{d=1}^{D} {y_d}^2}} \tag{17}$$

where x_d and y_d are the dth components of vectors $\boldsymbol{x}$ and $\boldsymbol{y}$, respectively.

For each wiring PI gradient, we computed the cosine similarity between its spatial distribution and the expression pattern of each gene across the 213 brain regions. Similarity scores were evaluated using the absolute value of cosine similarity to assess the strength of spatial correspondence, while the sign of the original score was retained to distinguish between positive and negative associations.

Spatial null maps of gene expression

We generated spatial null maps that preserve the intrinsic spatial autocorrelation of regional gene expression using BrainSMASH(63). BrainSMASH first estimates the empirical variogram of each spatial gene-expression profile $\boldsymbol{x}_g \in \mathbb{R}^{N_{\text{regions}}}$ using the inter-regional distance matrix $D \in \mathbb{R}^{N_{\text{regions}} \times N_{\text{regions}}}$, and then samples surrogate maps (i.e., randomized gene-expression patterns) whose spatial covariance is constrained to match the estimated variogram.

In this study, we generated 1,000 surrogate maps for each of the 763 genes. These surrogate maps were used to statistically evaluate the similarity of each gene's expression pattern to the wiring PI gradients. They also served as the basis for a spatial null model in which the spatial distribution of gene expression was randomized while preserving its intrinsic spatial autocorrelation.

Statistical evaluation of similarity between gene expression pattern and the wiring PI gradients

To assess whether the observed cosine similarity scores between each gene-expression pattern and the wiring PI gradients were significantly higher than expected by chance, we compared them

against null distributions derived from the corresponding surrogate maps. Specifically, for each gene, we computed similarity scores between its 1,000 surrogate maps and the wiring PI gradients, and calculated empirical p-values by comparing the observed similarity score with the null distribution. We further applied false discovery rate (FDR) correction to obtain q-values. Genes with $p < 0.05$ are listed in **Table S1** and **Table S2**.

Gene ontology (GO) enrichment analysis

To assess whether the identified gene sets were biased toward specific molecular functions or biological processes, we performed gene ontology (GO) enrichment analysis using g:Profiler(48). We first analyzed the full set of 763 genes used in this study and confirmed that they were enriched for neural- and development-related categories (SI appendix, **Fig. S6**). We then tested whether the genes showing high spatial similarity to each wiring PI component ($p < 0.05$) were enriched for any GO terms relative to the background set of all 763 genes. No significant enrichment was detected. We also performed GO enrichment on the combined gene set across all wiring PI components, but again observed no significant enrichment. The detailed analysis settings and results can be accessed via the URLs listed in **Table S3**.

Reconstruction of neural connection matrix

To evaluate the validity of the chemoaffinity theory in explaining neural circuit formation, we reconstructed the neural connectivity matrix using the wiring PI gradients identified by the SPERRFY framework. According to the chemoaffinity theory, the positions of axonal projections are determined by specific molecular concentration mappings between the source and target regions. In the framework of SPERRFY, these molecular mappings are represented by the proximity of the wiring PI values at the source and target regions. Notably, since the wiring PI gradients obtained through CCA are standardized and expressed in terms of positive correlation, comparing their magnitudes provides a reasonable measure of positional relationships.

First, we quantified the differences in the identified wiring PI values for each pair of brain regions. For the top five most correlated PI components $\mathrm{PI}_s{}^{(i)}(\boldsymbol{r}_s)$ and $\mathrm{PI}_t{}^{(i)}(\boldsymbol{r}_t)$, the L2 norm of their absolute differences was calculated as :

$$d_{PI}(\boldsymbol{r}_s, \boldsymbol{r}_t) = \frac{1}{Z}\sqrt{\sum_{\mathrm{i}=1}^{5}\left(\mathrm{PI}_s{}^{(i)}(\boldsymbol{r}_s) - \mathrm{PI}_t{}^{(i)}(\boldsymbol{r}_t)\right)^2} \tag{18}$$

where $\boldsymbol{x}_s$ and $\boldsymbol{x}_t$ represent the spatial coordinates of the source and target brain regions, respectively, and Z is the normalization term. $d_{PI}(\boldsymbol{r}_s, \boldsymbol{r}_t)$ is normalized such that its maximum value is set to 1, ensuring a consistent range for further analysis. A value closer to 0 in this matrix indicates a higher likelihood of neural connectivity.

Second, we reconstructed connectivity matrices by binarizing the $d_{PI}(\boldsymbol{r}_s, \boldsymbol{r}_t)$ matrix with a threshold, where region pairs with PI differences below the threshold were classified as connected. To generate the full receiver operating characteristic (ROC) curve, the decision threshold was systematically swept from 0 to 1 in 0.01 increments (101 operating points). The accuracy of the reconstructed connectivity was evaluated by comparing these thresholded matrices to the original connectome. The performance was assessed using the area under the curve (AUC), providing a quantitative measure of how well the reconstructed matrices predict actual neural connections.

Null models for evaluating wiring positional information

To verify whether the wiring PI identified by SPERRFY reflects biologically meaningful connectivity patterns rather than occurring by chance, we performed statistical testing by employing two types of null models: the globally randomized model and the locally randomized model.

In the globally randomized model, connections were randomly reassigned across the entire connectivity matrix (without any structural constraints), while preserving the total number of neural connections in the original connectome (**Fig. 6A, left**). This approach generated connectivity matrices where the connections were uniformly distributed across all brain regions. In contrast, the locally randomized model imposed structural constraints by preserving not only the total number of

neural connections but also the number of connections between MRs (**Fig. 6A, right**). Specifically, connections were randomly reassigned within each MR pair while maintaining the original connection counts for each pair, thereby retaining macroscale connectivity patterns such as inter-region projection density.

To further refine the analysis, two additional constraints were individually applied to the null models in order to better reflect key features of the mouse brain connectome. First, a constraint was introduced to preserve the connection distance distribution by utilizing data from Oh et al., 2014(24), which provides detailed information on the spatial organization of neural connections in the mouse brain. Connection distances were binned into histograms with a bin width of 100 μm (SI appendix, **Fig. S12**), and connections were randomly reassigned while maintaining the original distribution across these bins. Second, another constraint was applied to preserve network topology by generating randomized connectivity matrices through shuffling the order of brain region annotations while maintaining the original connection patterns. This procedure retained important topological features of the network, such as degree distribution and hub structures, while randomizing the association between connectivity patterns and gene expression profiles.

For each null model, 1,000 randomized connectivity matrices were generated, and SPERRFY was applied to extract wiring PI gradients from each instance. Subsequently, the empirical null distribution of the correlation coefficients between the extracted PI gradients and the accuracy of the reconstructed connectivity was obtained. Statistical significance was assessed by comparing these null distributions with those derived from the original connectome data.

We also evaluated the effect of bin width in the distance-preserving constraint by testing additional bin sizes of 50 μm and 200 μm, in addition to the default 100 μm (SI appendix, **Fig. S13**). In the globally randomized model, the results were largely consistent across different bin widths, indicating that these settings effectively randomized connectivity while preserving distance dependence. In contrast, the locally randomized model showed higher performance when a smaller bin width was used. Given the inter-regional distance distributions for each MR pair (SI appendix, **Fig. S12B**), this effect is likely due to the finer binning producing distance histograms that more closely approximate those of the original data, thereby generating randomized connectivity patterns that are more similar to the empirical connectome.

Evaluation of the similarity of wiring PI gradients

To evaluate the similarity between wiring PI gradients extracted from the null-model data and those obtained from the original connectome data, we quantified the pairwise similarity as follows:

$$S_{\mathrm{PI}}\left(\mathbf{PI}_{orig}{}^{(i)}, \mathbf{PI}_{null}{}^{(i)}\right) = \frac{1}{2}\left|S_C\left(\mathrm{PI}_{orig,s}{}^{(i)}, \mathrm{PI}_{null,s}{}^{(i)}\right) + S_C\left(\mathrm{PI}_{orig,t}{}^{(i)}, \mathrm{PI}_{null,t}{}^{(i)}\right)\right| \quad (19)$$

where $\mathbf{PI}_{orig}{}^{(i)} = \left(\mathrm{PI}_{orig,s}{}^{(i)}, \mathrm{PI}_{orig,t}{}^{(i)}\right)$ and $\mathbf{PI}_{null}{}^{(i)} = \left(\mathrm{PI}_{null,s}{}^{(i)}, \mathrm{PI}_{null,t}{}^{(i)}\right)$ are the ith pair of wiring PI vectors extracted from original connectome data and null-model data, respectively, and $S_C(\boldsymbol{x}, \boldsymbol{y})$ denotes cosine similarity defined in Eq. (17). A higher value of S_{PI} indicates greater similarity between the null model-derived wiring PI and that from the original data.

Null model analysis using spatially randomized gene-expression patterns

To evaluate whether the wiring PI gradients identified by SPERRFY reflected biological structure beyond spatial autocorrelation, we constructed a null model in which the spatial distributions of gene expression were randomized while preserving their intrinsic spatial autocorrelation. Spatially randomized gene-expression patterns were generated using the spatial null maps produced by BrainSMASH (see *"Spatial null maps of gene expression"*)(63). For each of the 763 genes, 1,000 spatial null maps were generated, yielding 1,000 spatially randomized gene-expression datasets. Each dataset was processed identically to the real data: expression values were concatenated across genes and reduced to 50 principal components by PCA, followed by SPERRFY to extract wiring PI gradients.

For each null dataset, we computed the correlation between the resulting and real PI gradients, as well as the AUC for connectivity reconstruction. The null distributions of these metrics were then compared with those from the real data to assess significance.

Figures

A

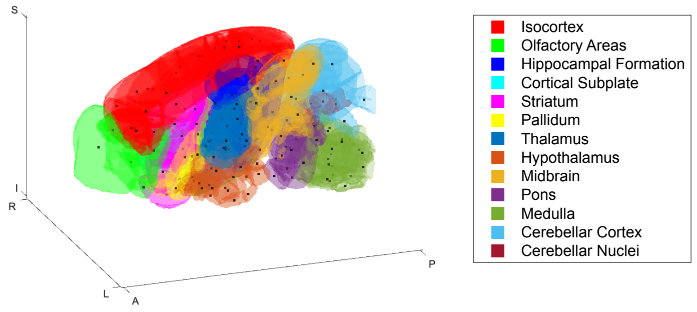

B

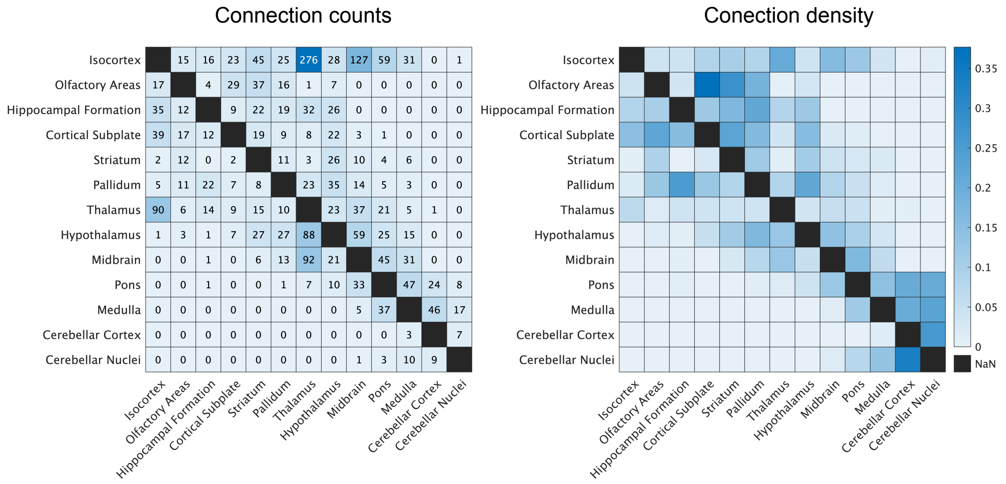

Fig. S1. Properties of the major regions (MRs) in the mouse brain.

A: 3D spatial visualization of the 13 major regions (MRs) in the mouse brain.
B: (Left) Number of connections between each MR pair. (Right) Connection density normalized by the number of possible connections.

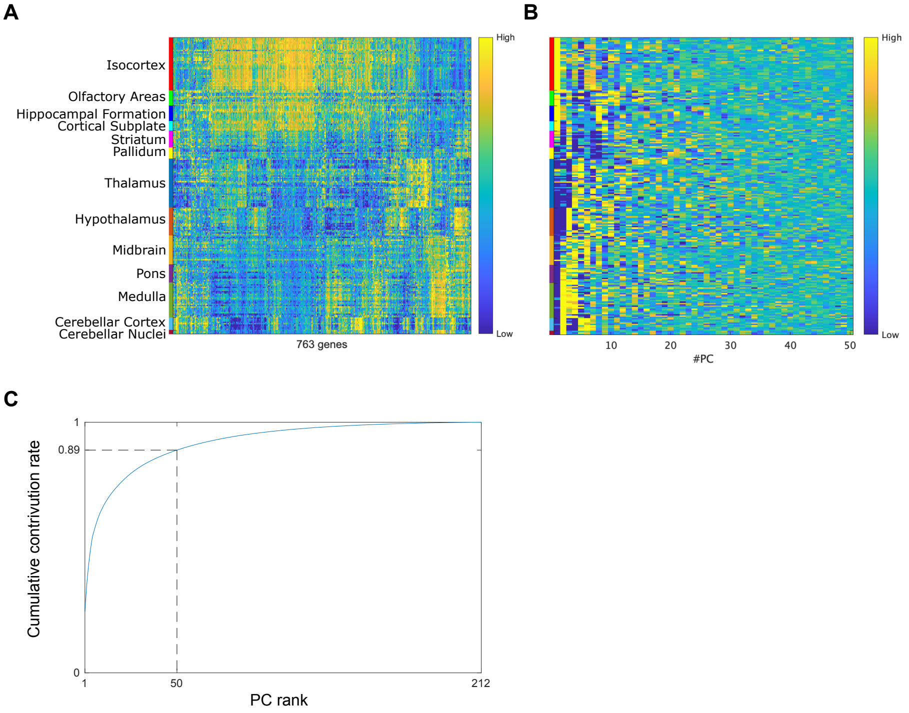

Fig. S2. Overview of gene expression data in the mouse brain.

A: Gene expression matrix showing normalized expression values for 763 genes across 213 brain regions.

B: Heatmap of the PCA-reduced gene expression data, showing the expression profiles of 213 brain regions across the top 50 principal components (PCs). Color represents expression values.

C: Cumulative contribution ratio of the top principal components. The dashed line indicates the 50th principal component, which was used as the dimensionality cutoff in the analysis.

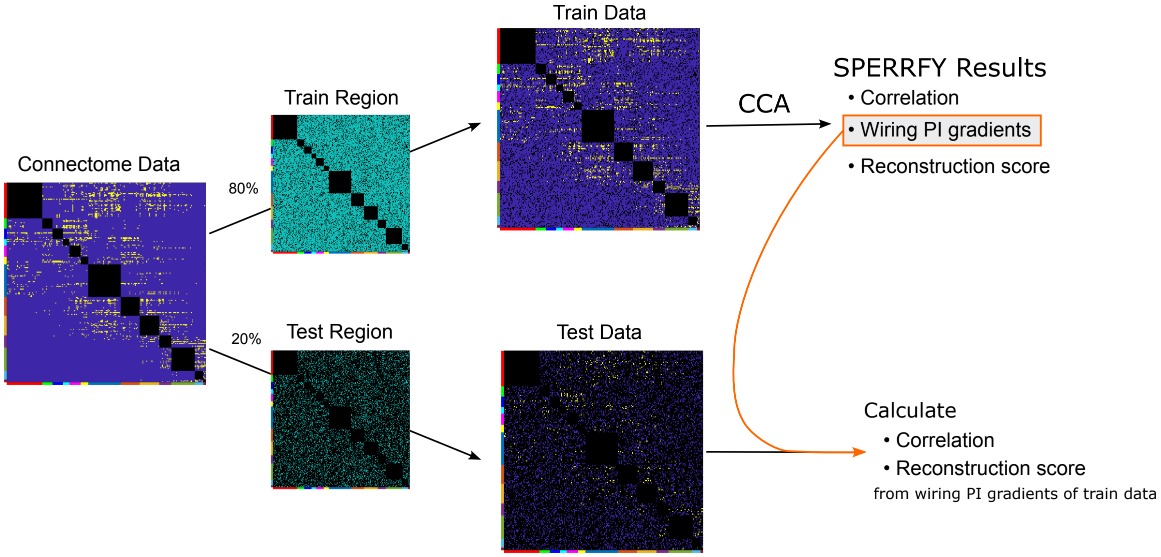

Fig. S3. Schematic illustration of the hold-out validation procedure.

The original connectome matrix was randomly split into a training region and a test region. CCA was applied only to the training data to extract wiring PI gradients. These gradients were then used to compute correlation coefficients and reconstruction performance on the held-out test data, thereby assessing the generalizability of SPERRFY results.

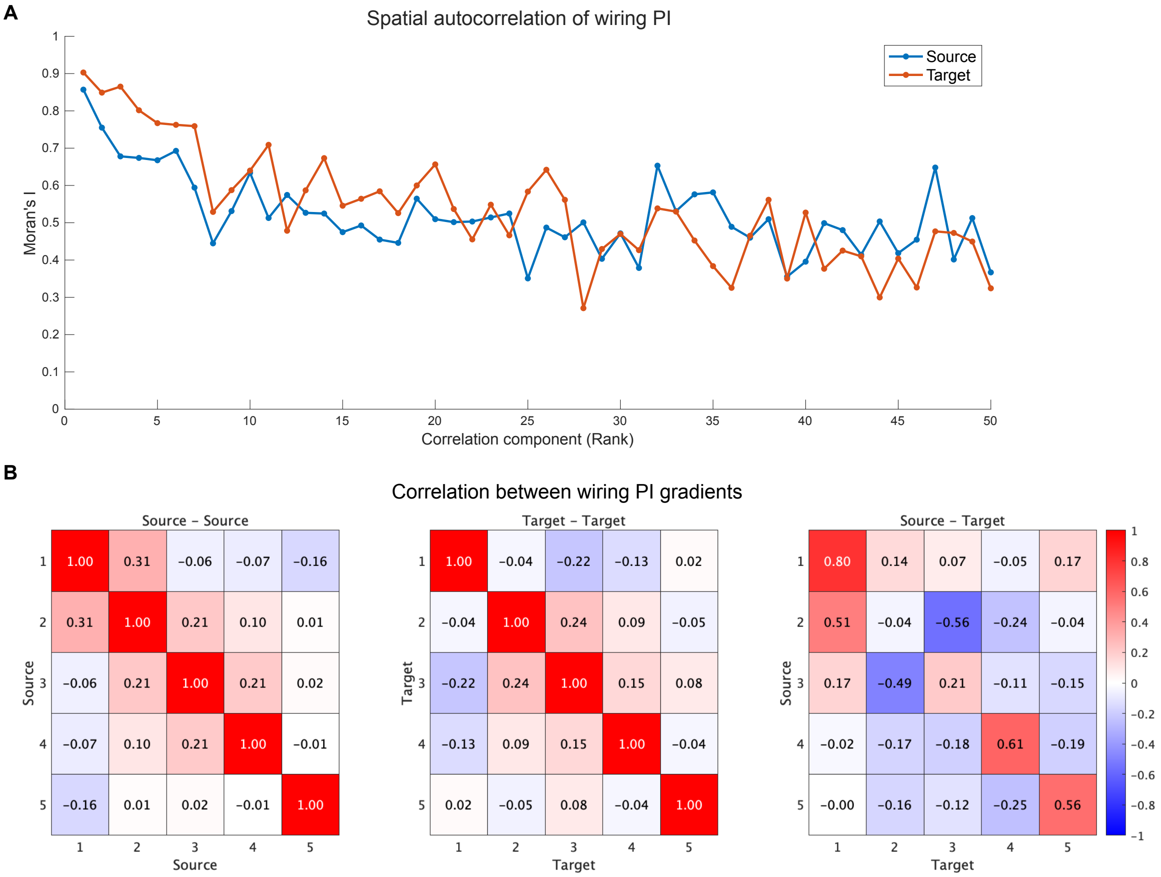

Fig. S4. Spatial autocorrelation and gradient correlations of wiring PI.

A: Spatial autocorrelation of each wiring PI component. Blue line indicates source wiring PI, and red line indicates target wiring PI. Spatial autocorrelation was quantified using Moran's I.

B: Correlations between wiring PI gradients. Shown are correlations between source–source (left), target–target (middle), and source–target (right) components.

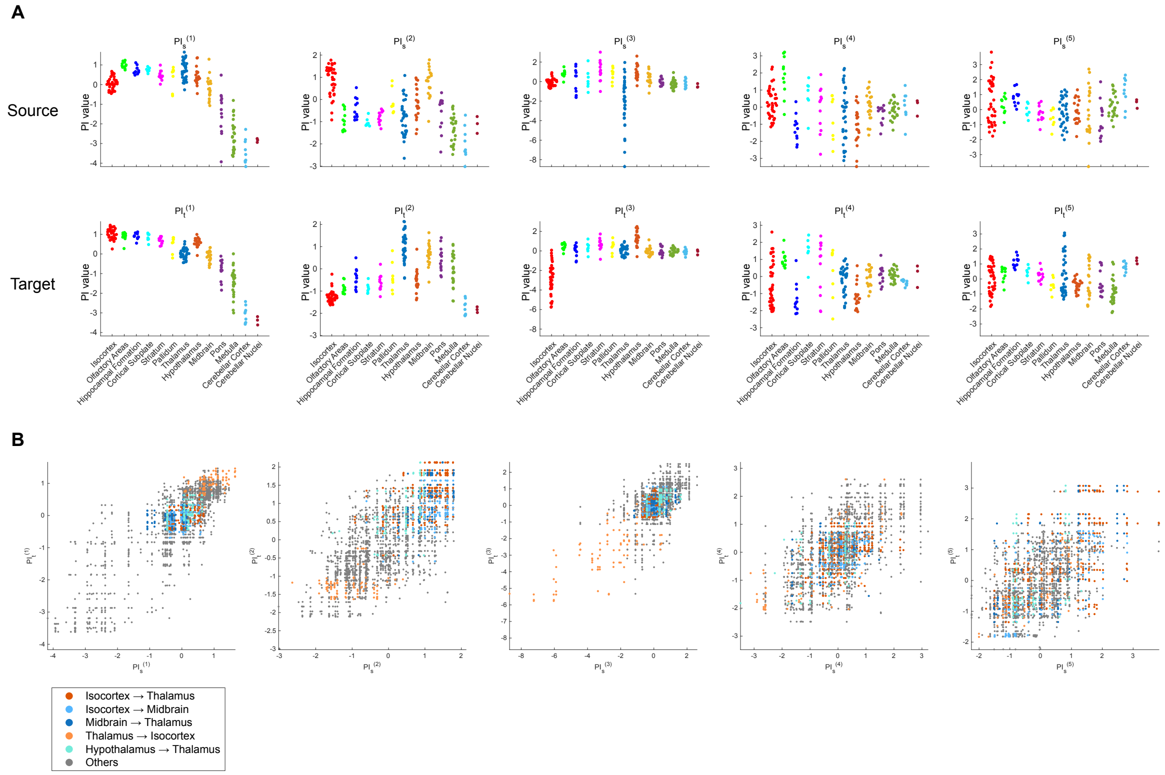

Fig. S5. Properties of wiring positional information (PI) for major regions (MRs).

A: Distribution of wiring PI values across brain regions, grouped by MR. Each dot represents a single brain region. Top: source PI components. Bottom: target PI components.

B: Scatter plots of wiring PI components, in which the five MR pairs with the highest numbers of inter-regional connections are color-coded, and all remaining pairs are shown in gray ("Others"). The plots correspond to Figure 3C but with different color coding.

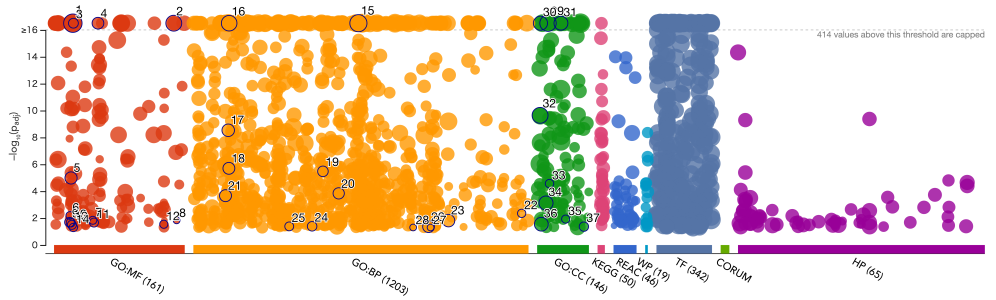

ID	Source	Term ID	Term Name	p_{adj} (query_1)
1	GO:MF	GO:0005515	protein binding	3.702×10^{-85}
2	GO:MF	GO:0140110	transcription regulator activity	1.708×10^{-55}
3	GO:MF	GO:0008066	glutamate receptor activity	1.710×10^{-17}
4	GO:MF	GO:0019199	transmembrane receptor protein kinase activity	3.620×10^{-17}
5	GO:MF	GO:0005201	extracellular matrix structural constituent	1.028×10^{-5}
6	GO:MF	GO:0005042	netrin receptor activity	6.364×10^{-3}
7	GO:MF	GO:0016907	G protein-coupled acetylcholine receptor activ...	1.449×10^{-2}
8	GO:MF	GO:0140788	L-glutamate uniporter activity	1.472×10^{-2}
9	GO:MF	GO:0004993	G protein-coupled serotonin receptor activity	1.999×10^{-2}
10	GO:MF	GO:0005161	platelet-derived growth factor receptor binding	2.146×10^{-2}
11	GO:MF	GO:0017154	semaphorin receptor activity	2.146×10^{-2}
12	GO:MF	GO:0098821	BMP receptor activity	2.828×10^{-2}
13	GO:MF	GO:0005326	neurotransmitter transmembrane transporter a...	3.138×10^{-2}
14	GO:MF	GO:0008046	axon guidance receptor activity	4.969×10^{-2}
15	GO:BP	GO:0048731	system development	1.026×10^{-138}
16	GO:BP	GO:0007267	cell-cell signaling	2.822×10^{-62}
17	GO:BP	GO:0007156	homophilic cell adhesion via plasma membrane...	2.916×10^{-9}
18	GO:BP	GO:0007218	neuropeptide signaling pathway	1.977×10^{-6}
19	GO:BP	GO:0042417	dopamine metabolic process	3.317×10^{-6}
20	GO:BP	GO:0045600	positive regulation of fat cell differentiation	1.442×10^{-4}
21	GO:BP	GO:0006835	dicarboxylic acid transport	2.138×10^{-4}
22	GO:BP	GO:2000677	regulation of transcription regulatory region D...	4.416×10^{-3}
23	GO:BP	GO:0141193	nuclear receptor-mediated signaling pathway	1.531×10^{-2}
24	GO:BP	GO:0035864	response to potassium ion	4.050×10^{-2}
25	GO:BP	GO:0032341	aldosterone metabolic process	4.108×10^{-2}
26	GO:BP	GO:0097120	receptor localization to synapse	4.313×10^{-2}
27	GO:BP	GO:0097477	lateral motor column neuron migration	4.961×10^{-2}
28	GO:BP	GO:0072278	metanephric comma-shaped body morphogen...	4.961×10^{-2}
29	GO:CC	GO:0030054	cell junction	8.422×10^{-81}
30	GO:CC	GO:0005667	transcription regulator complex	1.104×10^{-24}
31	GO:CC	GO:0043235	receptor complex	1.978×10^{-22}
32	GO:CC	GO:0005615	extracellular space	2.267×10^{-10}
33	GO:CC	GO:0030877	beta-catenin destruction complex	2.691×10^{-5}
34	GO:CC	GO:0015629	actin cytoskeleton	7.564×10^{-4}
35	GO:CC	GO:0060198	clathrin-sculpted vesicle	1.181×10^{-2}
36	GO:CC	GO:0005769	early endosome	2.834×10^{-2}
37	GO:CC	GO:1902711	GABA-A receptor complex	4.289×10^{-2}

**version**	e113_eg59_p19_f6a03c19
**date**	2025/11/12 11:59:45
**organism**	mmusculus

g:Profiler

Fig. S6. Gene ontology (GO) enrichment analysis of the full set of 763 genes used in this study.

GO enrichment analysis was performed using g:Profiler to evaluate whether the full set of 763 genes included in this study shows bias toward specific molecular functions, biological processes, or cellular components. The analysis revealed significant enrichment in several neural- and development-related categories. Detailed settings and the complete results can be accessed via the URLs listed in Table S3.

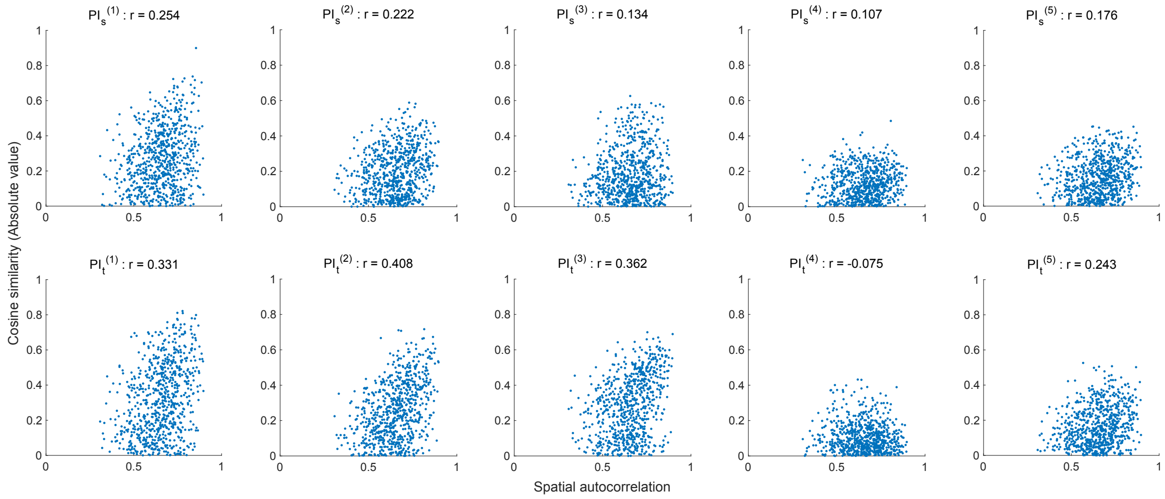

Fig. S7. Relationship between cosine similarity and spatial autocorrelation of each gene. Absolute cosine similarity to the wiring PI gradients is plotted against spatial autocorrelation (Moran's I). Only weak correlations are observed, indicating that top-ranked genes are not simply those with smooth spatial profiles.

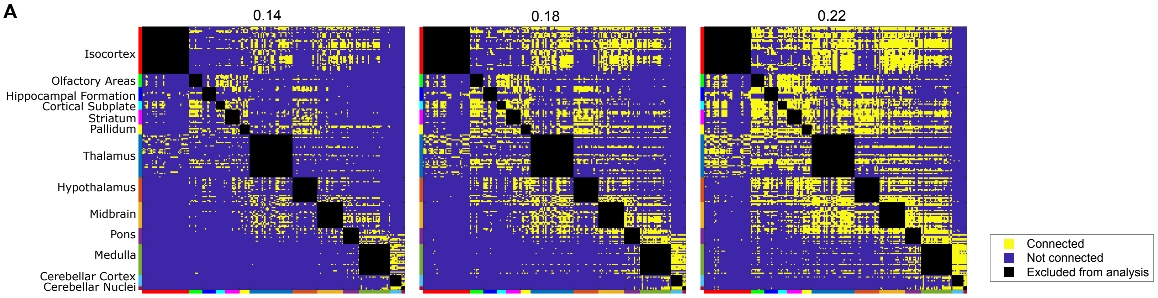

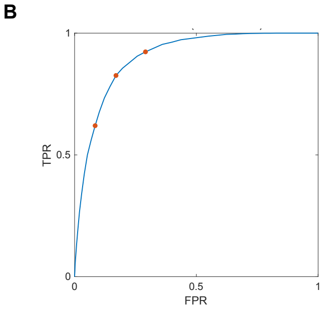

Fig. S8. Examples of connection matrices reconstructed using wiring PI.

A: Reconstructed binary connection matrices obtained using threshold values of 0.14, 0.18, and 0.22, based on wiring PI derived from the full-data analysis.

B: Receiver operating characteristic (ROC) curve for connection reconstruction. Red dots correspond to the three threshold conditions used in (A).

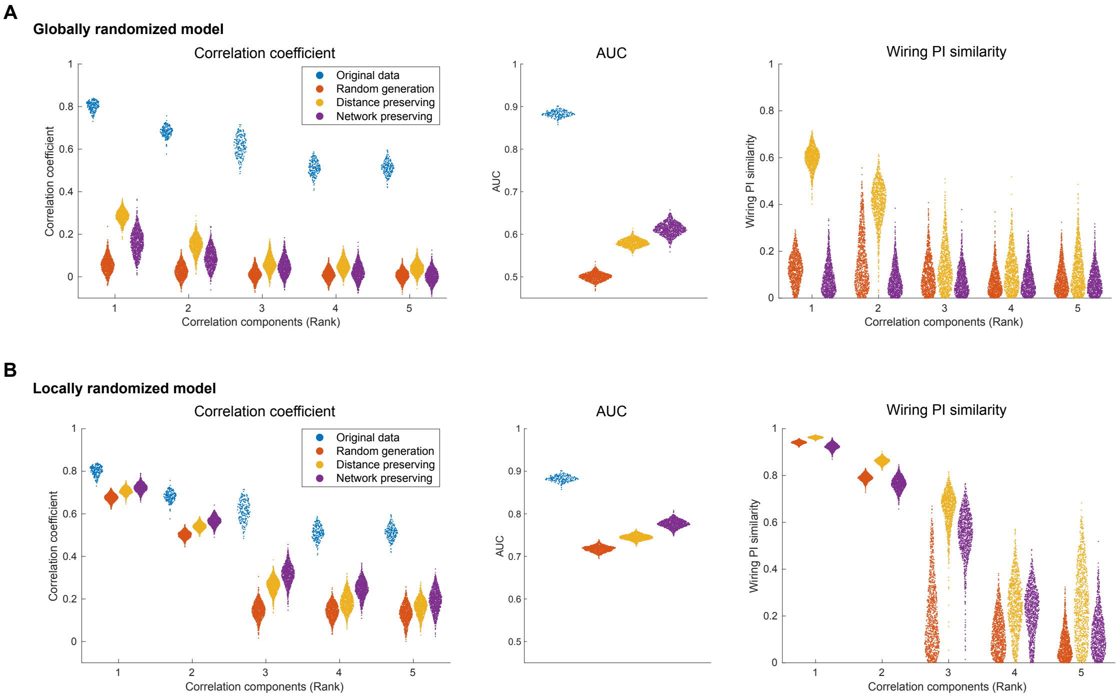

Fig. S9. Comparison with additional null models incorporating biological constraints.

A: Results from the globally randomized models with additional constraints. (Left) Distributions of the top five correlation coefficients extracted from each dataset. (Middle) Distributions of AUC values for reconstructed connectivity across the original and randomized models. (Right) Similarity scores between the wiring PI gradients of the randomized and original data. Color coding is shown in the left panel. Correlation coefficients and AUCs are evaluated on held-out datasets not used for CCA parameter estimation.

B: Results from the locally randomized model with additional constraints. The panel layout and color coding are consistent with (A). Correlation coefficients and AUCs are evaluated on held-out datasets not used for CCA parameter estimation.

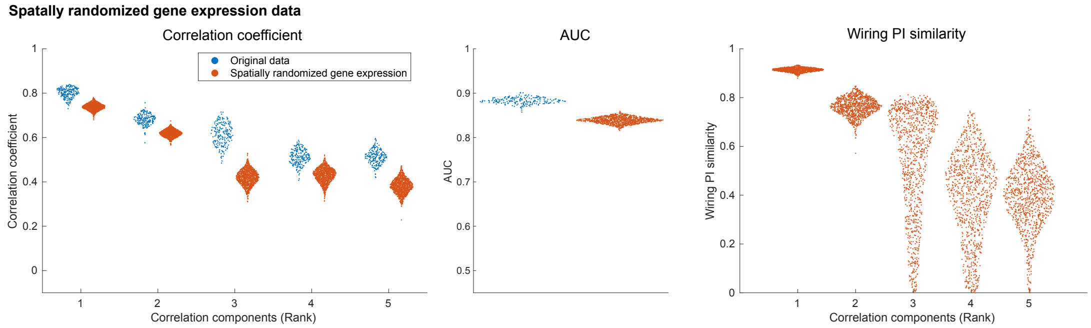

Fig. S10. Results using spatially randomized gene expression data.

Analyses performed on spatially randomized gene-expression datasets, shown in comparison with results from the original connectome. (Left) Distributions of the top five correlation coefficients extracted from each dataset. (Middle) Distributions of AUC values for reconstructed connectivity across the original and randomized models. (Right) Similarity scores between the wiring PI gradients of the randomized and original data. Color coding is shown in the left panel. Correlation coefficients and AUCs are evaluated on held-out datasets not used for CCA parameter estimation.

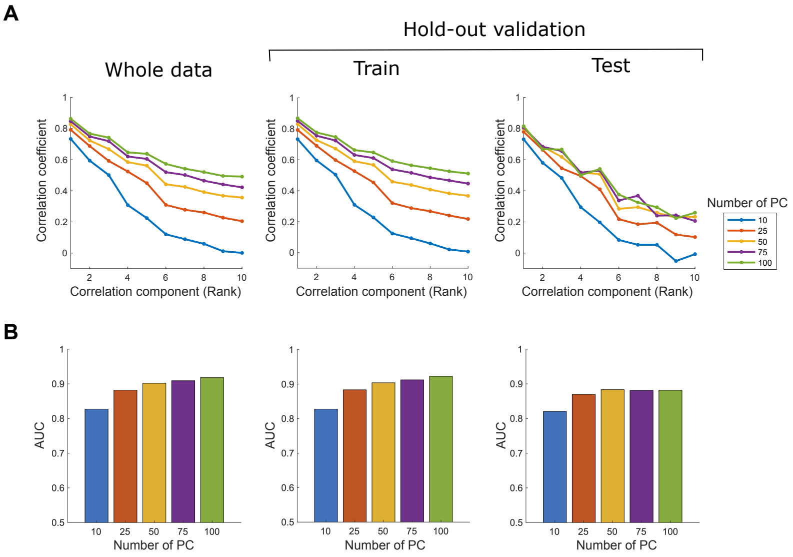

Fig. S11. Evaluation of the effect of PCA dimensionality on SPERRFY performance.

A: Correlation coefficients of the top 10 wiring PI components across different numbers of principal components (PCs) used for gene expression representation (10, 25, 50, 75, 100).
B: AUC scores for connectome reconstruction evaluated on the whole dataset and under hold-out validation (train/test split).

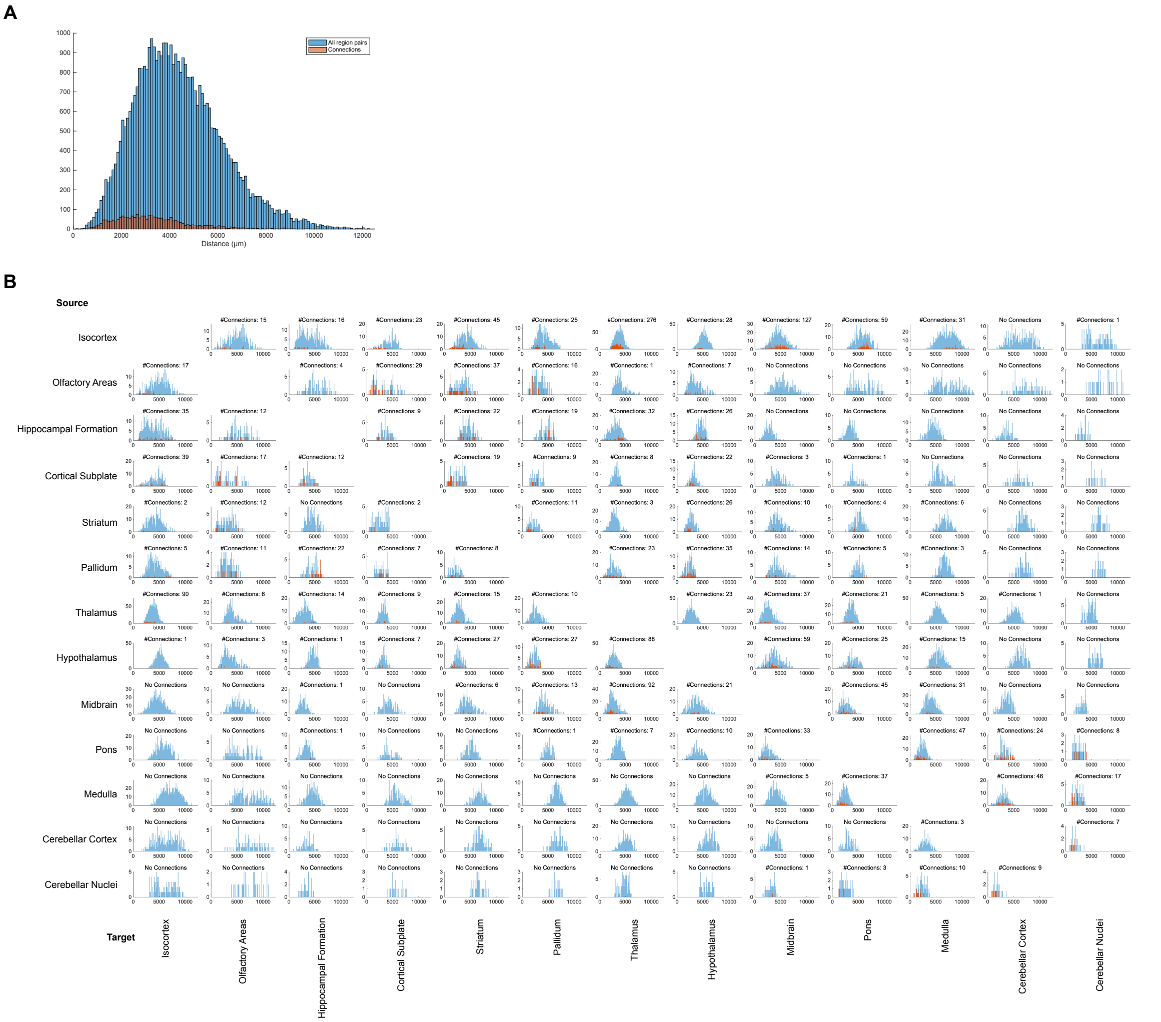

Fig. S12. Histograms of inter-regional distances used to construct the distance-preserving null model.

A: Histogram of inter-regional distances. Each count corresponds to one directed (source–target) region pair. Blue bins represent all region pairs, and red bins represent connected region pairs. The bin width is 100 μm.

B: Histograms of inter-regional distances plotted separately for each directed MR pair.

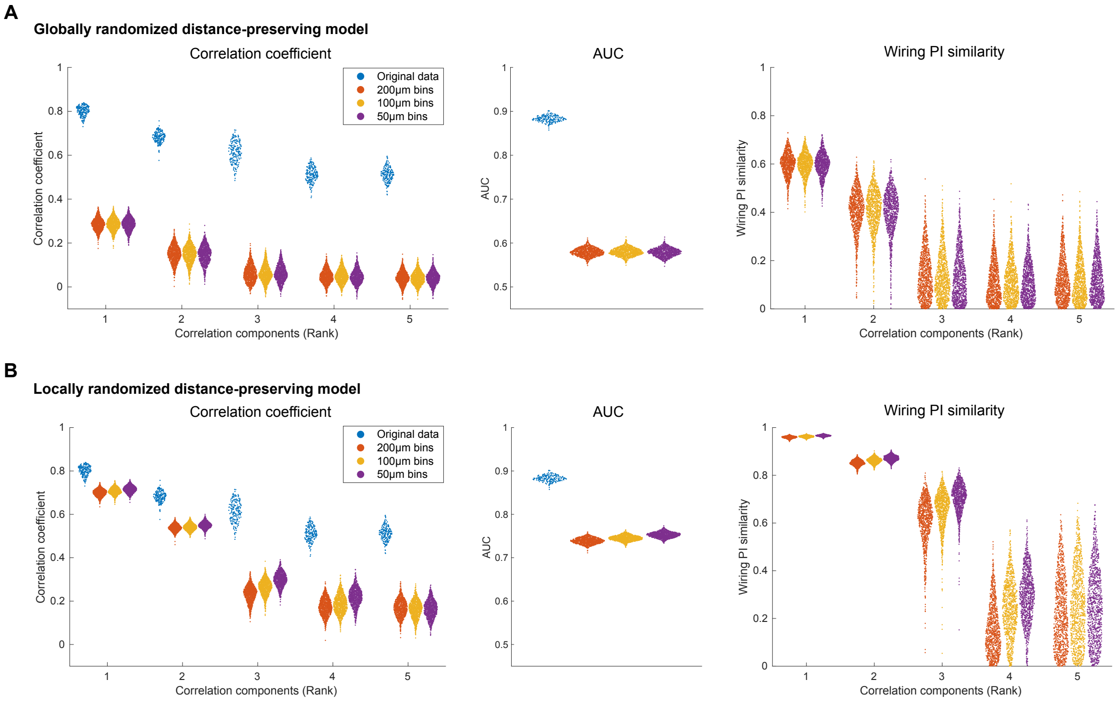

Fig. S13. Comparison of distance-preserving null models with different bin widths.

A: Results from the globally randomized distance-preserving models using different bin widths. (Left) Distributions of the top five correlation coefficients extracted from each dataset. (Middle) Distributions of AUC values for reconstructed connectivity across the original and randomized models. (Right) Similarity scores between the wiring PI gradients of the randomized and original data. Color coding is shown in the left panel. Correlation coefficients and AUCs are evaluated on held-out datasets not used for CCA parameter estimation.

B: Results from the locally randomized distance-preserving models using different bin widths. The panel layout and color coding are consistent with (A). Correlation coefficients and AUCs are evaluated on held-out datasets not used for CCA parameter estimation.

Tables

	PI source 1				PI source 2				PI source 3				PI source 4				PI source 5			
Rank	Gene	Sim	*p*	*q*	Gene	Sim	*p*	*q*	Gene	Sim	*p*	*q*	Gene	Sim	*p*	*q*	Gene	Sim	*p*	*q*
1	Cpne7	0.90	0.001	0.25	Ephb6	0.59	0.001	0.16	Rgs10	0.63	0.001	0.01	Robo2	0.48	0.001	0.25	Boc	0.45	0.001	0.76
2	Nrg1	-0.74	0.001	0.25	Adcy2	0.58	0.001	0.16	Parm1	0.59	0.001	0.01	Slc2a1	-0.42	0.001	0.25	Abca2	-0.45	0.013	0.85
3	Lhx1	-0.73	0.006	0.46	Chrm3	0.56	0.001	0.16	Plekhg1	-0.58	0.001	0.01	C1ql2	-0.40	0.002	0.30	Mxi1	0.45	0.008	0.85
4	Pdgfra	0.72	0.005	0.46	Cbln2	0.55	0.002	0.16	Zmat4	-0.58	0.001	0.01	Tshz1	0.38	0.001	0.25	Nr4a3	0.45	0.004	0.85
5	Camk2a	0.72	0.011	0.64	Lrp1	0.55	0.001	0.16	Nnat	0.58	0.001	0.01	Zfp462	0.35	0.004	0.30	Ache	-0.43	0.018	0.85
6	Ly6h	0.71	0.003	0.46	Zic1	-0.53	0.004	0.18	Kcnj12	-0.57	0.001	0.01	Mapk8	0.35	0.007	0.31	Loxl1	0.43	0.004	0.85
7	Loxl2	-0.70	0.001	0.25	Atp8a1	0.53	0.002	0.16	Peg10	0.57	0.001	0.01	Zic1	-0.33	0.006	0.31	Neurod1	0.43	0.019	0.85
8	Pcdh1	0.69	0.024	0.82	Arnt2	0.51	0.002	0.16	Lef1	-0.56	0.001	0.01	Pax8	0.33	0.007	0.31	Slc18a2	-0.42	0.021	0.85
9	Ctnnb1	0.68	0.007	0.49	Hopx	-0.50	0.002	0.16	Adcyap1r1	0.56	0.001	0.01	Gsk3a	0.33	0.002	0.30	Col5a1	0.42	0.019	0.85
10	Grm5	0.68	0.018	0.72	L3mbtl1	0.50	0.003	0.16	Hap1	0.56	0.001	0.01	Cux2	0.32	0.004	0.30	Fezf2	0.42	0.034	0.85
11	Cacna2d1	0.68	0.005	0.46	Plcxd2	0.49	0.006	0.18	Chl1	0.56	0.001	0.01	Tpd52l1	-0.32	0.008	0.31	Hoxd3	0.41	0.022	0.85
12	Kctd9	-0.67	0.003	0.46	Nde1	0.49	0.002	0.16	Tcerg1l	0.56	0.001	0.01	Ece2	-0.32	0.011	0.31	Lhx2	0.41	0.021	0.85
13	Astn1	0.67	0.015	0.71	Robo2	0.49	0.005	0.18	Kcnc2	-0.55	0.001	0.01	Nrp1	-0.31	0.007	0.31	Nfil3	0.41	0.011	0.85
14	Cyc1	0.65	0.017	0.72	Dkk3	0.48	0.006	0.18	Setd7	-0.54	0.001	0.01	Glp1r	-0.31	0.013	0.31	Pde5a	0.41	0.028	0.85
15	Top1	0.65	0.006	0.46	Mapk8ip3	0.48	0.003	0.16	Gja1	0.54	0.001	0.01	Hoxd3	0.31	0.021	0.33	Hes3	0.40	0.033	0.85
16	Grin2b	0.65	0.008	0.51	Nrgn	0.47	0.008	0.18	Cnr1	0.53	0.001	0.01	Epha5	0.31	0.003	0.30	Baiap3	-0.39	0.030	0.85
17	Cdh2	0.65	0.004	0.46	Snw1	0.47	0.005	0.18	Nos1	0.53	0.001	0.01	Zfhx3	-0.30	0.015	0.31	Robo1	-0.39	0.022	0.85
18	Foxb1	-0.64	0.034	0.86	Kcnk2	0.47	0.012	0.25	Pcdh11x	0.53	0.001	0.01	Plxnd1	0.30	0.018	0.33	Col1a1	0.39	0.019	0.85
19	Pcdh19	0.63	0.026	0.82	Slitrk1	0.47	0.004	0.18	Trhr	0.52	0.001	0.01	Jazf1	0.30	0.011	0.31	Dlk1	-0.39	0.032	0.85
20	Zfp521	-0.63	0.014	0.71	Dach1	-0.47	0.011	0.24	Unc5c	-0.51	0.001	0.01	Scube1	0.30	0.006	0.31	Mybpc1	0.39	0.041	0.85
21	Nxph4	-0.63	0.030	0.85	Fam3c	0.47	0.017	0.29	Irs4	0.51	0.001	0.01	Srebf2	0.30	0.014	0.31	Abat	-0.39	0.026	0.85
22	Necab2	0.62	0.017	0.72	Pvrl3	0.47	0.006	0.18	Grin2c	-0.51	0.001	0.01	Atp1a2	-0.30	0.005	0.31	Nfix	0.39	0.050	0.85
23	Cdh8	0.61	0.022	0.82	Psd	0.47	0.006	0.18	Psd	0.51	0.001	0.01	Dab1	0.30	0.020	0.33	Glcci1	0.39	0.019	0.85
24	Grid1	0.61	0.012	0.65	Fam189a2	-0.46	0.003	0.16	Mgst3	-0.50	0.002	0.02	Spint2	-0.29	0.019	0.33	Lmx1a	0.38	0.027	0.85
25	Zfp207	0.61	0.029	0.85	Cdh11	0.46	0.006	0.18	Scg3	0.50	0.002	0.02	Egr2	0.29	0.023	0.35	Prox1	0.38	0.040	0.85
26	Syn1	0.60	0.027	0.82	Mpped1	0.45	0.018	0.29	Inpp4b	-0.50	0.001	0.01	Ddr1	-0.29	0.016	0.31	Tcf4	0.38	0.050	0.85
27	Gria1	0.59	0.043	0.96	Gabrg2	0.45	0.008	0.18	Grm1	-0.50	0.001	0.01	Ache	-0.29	0.013	0.31	Clcn5	-0.37	0.048	0.85
28	Spp1	-0.59	0.027	0.82	Tle4	0.45	0.003	0.16	Nefh	-0.50	0.001	0.01	Grm8	0.29	0.010	0.31	Eya4	0.37	0.005	0.85
29	Pvalb	-0.58	0.025	0.82	Cacna2d1	0.45	0.010	0.22	Slit1	0.50	0.001	0.01	Hspb8	-0.29	0.014	0.31	Zfpm2	0.37	0.042	0.85
30	Apc	0.57	0.038	0.91	Egr1	0.45	0.008	0.18	C230009H10Rik	-0.50	0.001	0.01	Stxbp6	0.29	0.024	0.35	Rps12	-0.37	0.041	0.85
31	Gap43	0.57	0.032	0.86	Nr2c2	0.45	0.007	0.18	Tpd52l1	-0.50	0.001	0.01	Fstl1	-0.29	0.012	0.31	Arntl	0.36	0.040	0.85
32	Cacna1h	0.57	0.046	0.98	Zdhhc2	0.45	0.007	0.18	Mesdc2	0.50	0.001	0.01	Mylk	-0.29	0.008	0.31	Bmpr1a	0.36	0.037	0.85
33	Pcsk2	0.56	0.042	0.96	Pou6f1	0.44	0.015	0.29	Amotl1	-0.49	0.002	0.02	Zkscan6	0.29	0.030	0.37	Igfbp5	0.36	0.046	0.85
34	Esrrg	-0.55	0.049	0.98	Col9a3	-0.44	0.008	0.18	Alcam	0.48	0.001	0.01	Gfra1	-0.28	0.014	0.31	Bmp5	0.36	0.025	0.85
35	Scg5	0.54	0.048	0.98	Erbb4	0.44	0.007	0.18	Calcr	0.48	0.003	0.03	Parvb	-0.28	0.012	0.31	Gli3	0.36	0.034	0.85
36	Col4a5	-0.52	0.034	0.86	Rprm	0.43	0.018	0.29	Ctbp2	0.48	0.001	0.01	Dbi	-0.28	0.011	0.31	Vat1	-0.35	0.044	0.85
37	Rnf14	0.52	0.036	0.89	Myt1	-0.43	0.024	0.35	Arl10	0.48	0.001	0.01	Ndnf	0.28	0.033	0.38	Jag1	0.35	0.017	0.85
38					Cdh24	0.43	0.007	0.18	Erbb4	0.48	0.001	0.01	Plcxd2	0.28	0.030	0.37	Ddc	-0.35	0.050	0.85
39					Grm5	0.43	0.018	0.29	Cit	-0.48	0.001	0.01	Aldh2	-0.28	0.011	0.31	Fmo1	0.34	0.020	0.85
40					Mapt	0.43	0.018	0.29	Prkcd	-0.47	0.001	0.01	Cd9	-0.28	0.016	0.31	Mylk	0.33	0.034	0.85
41					Lrp6	0.43	0.013	0.27	Ly6h	0.47	0.001	0.01	Zfp521	-0.28	0.030	0.37	Atf5	0.32	0.038	0.85
42					Prkcd	-0.43	0.018	0.29	Cartpt	0.47	0.001	0.01	Mgat5b	0.28	0.040	0.39	Trp53	0.32	0.046	0.85
43					Dvl1	0.43	0.014	0.28	Slc6a1	0.46	0.002	0.02	Mybl2	0.28	0.011	0.31	C1qb	0.31	0.032	0.85
44					Satb1	0.42	0.027	0.35	Atp8a1	0.46	0.001	0.01	Sparc	-0.27	0.021	0.33				
45					Mib2	0.42	0.003	0.16	Plcb4	-0.46	0.005	0.04	Maz	0.27	0.021	0.33				
46					Inpp4b	-0.42	0.036	0.38	Vat1l	0.46	0.001	0.01	Zbtb7a	0.27	0.016	0.31				
47					Plcg1	0.42	0.020	0.31	Gad2	0.46	0.001	0.01	Sertad2	0.27	0.019	0.33				
48					Egr3	0.41	0.039	0.40	Mest	0.45	0.003	0.03	Zfp516	0.27	0.023	0.35				
49					Zeb2	0.41	0.032	0.38	Slc32a1	0.45	0.001	0.01	Pcdhb9	0.27	0.038	0.39				
50					Gabrb2	0.41	0.021	0.31	Ndst4	0.45	0.002	0.02	Pcdh10	0.27	0.031	0.38				
51					Ddit4l	0.41	0.027	0.35	Ntng1	-0.44	0.003	0.03	Creb3	0.27	0.014	0.31				
52					Zbtb16	0.40	0.030	0.38	Gdf11	-0.44	0.003	0.03	S100a10	0.27	0.028	0.37				
53					Stxbp6	0.40	0.023	0.34	Trp53i11	0.44	0.001	0.01	Wnt9a	0.27	0.035	0.38				
54					Ptprk	0.40	0.027	0.35	Pcp4	-0.44	0.001	0.01	Nrg2	0.27	0.014	0.31				
55					Gfra2	0.40	0.008	0.18	Ctnnd1	-0.44	0.002	0.02	Jag1	0.27	0.011	0.31				
56					Nckap1	0.40	0.018	0.29	Efnb2	0.44	0.003	0.03	Fam189a2	-0.27	0.024	0.35				
57					Grik5	0.40	0.044	0.40	Nucb2	0.43	0.002	0.02	Ikzf5	0.27	0.039	0.39				
58					Astn1	0.40	0.025	0.35	Cntnap4	0.43	0.001	0.01	Calcr	-0.26	0.049	0.40				
59					Lmo3	0.40	0.036	0.38	Tcf7l2	-0.43	0.002	0.02	Sel1l	0.26	0.004	0.30				
60					Gria2	0.40	0.049	0.43	Grm4	-0.43	0.004	0.04	Bcar1	0.26	0.003	0.30				
61					Igfbp4	0.39	0.043	0.40	Aplp1	0.42	0.002	0.02	Gpr56	-0.26	0.034	0.38				
62					Alcam	0.39	0.031	0.38	Postn	0.42	0.001	0.01	Clcn5	-0.26	0.035	0.38				
63					Srebf2	0.39	0.021	0.31	Reln	0.42	0.008	0.06	Prox1	-0.26	0.030	0.37				
64					Rnf14	0.39	0.017	0.29	Fgfr1	0.42	0.002	0.02	Itgb8	0.26	0.035	0.38				
65					Zfp521	-0.38	0.038	0.39	Ecel1	0.42	0.001	0.01	Plxna2	0.26	0.020	0.33				
66					Maob	-0.38	0.031	0.38	Ets2	0.42	0.001	0.01	Mgst3	0.26	0.045	0.40				
67					Gsg1l	0.38	0.043	0.40	Pcdh19	0.42	0.003	0.03	Gpx3	-0.26	0.038	0.39				
68					Insrr	-0.38	0.033	0.38	Ahi1	0.42	0.004	0.04	Csnk1d	0.26	0.025	0.35				
69					Tcf25	0.38	0.006	0.18	Nts	0.42	0.001	0.01	Bcorl1	0.26	0.030	0.37				
70					Pomgnt1	0.38	0.043	0.40	Scn1a	-0.42	0.001	0.01	Fbxw7	0.26	0.045	0.40				
71					Lamb1	-0.37	0.020	0.31	Fam189a2	-0.41	0.001	0.01	Phf13	0.26	0.015	0.31				
72					Scg5	0.37	0.034	0.38	S100a10	0.41	0.004	0.04	Whrn	-0.26	0.047	0.40				
73					Top1	0.37	0.040	0.40	Tmed3	0.40	0.002	0.02	Pde10a	0.25	0.039	0.39				
74					Pou3f2	0.37	0.034	0.38	S1pr1	0.40	0.001	0.01	Mef2d	0.25	0.036	0.39				
75					Pik3r1	0.36	0.031	0.38	Ctnnbip1	-0.40	0.001	0.01	Tead3	-0.25	0.025	0.35				
76					Drg1	0.36	0.032	0.38	Spint2	0.40	0.002	0.02	Cdhr1	0.25	0.038	0.39				
77					Deaf1	0.36	0.034	0.38	Lamb1	-0.40	0.001	0.01	Sorl1	0.25	0.020	0.33				
78					Foxp1	0.35	0.046	0.41	Gabrb2	-0.40	0.004	0.04	Cdh4	0.24	0.026	0.36				
79					Pcsk2	0.35	0.048	0.43	Gpx3	0.40	0.007	0.06	Gabpb1	0.24	0.037	0.39				
80					Pcdh10	0.35	0.042	0.40	Grik3	0.39	0.005	0.04	Gsg1l	0.24	0.050	0.40				
81					Creb3	0.34	0.029	0.37	Rab3b	0.39	0.005	0.04	Zbtb7b	0.24	0.050	0.40				
82					Bcor	0.34	0.043	0.40	Tsc22d3	-0.39	0.001	0.01	Asic1	0.24	0.047	0.40				
83					Zscan26	0.34	0.038	0.39	Adcyap1	0.39	0.007	0.06	Qdpr	-0.24	0.044	0.40				
84					Sema6d	-0.33	0.041	0.40	Rps12	0.39	0.005	0.04	Pbxip1	-0.24	0.034	0.38				
85					Elf1	0.32	0.046	0.41	Gria1	0.39	0.010	0.07	Cst3	-0.24	0.050	0.40				
86					Mid1	0.32	0.036	0.38	Dlk1	0.38	0.010	0.07	Cadm3	0.23	0.049	0.40				
87					Eya4	0.31	0.026	0.35	Arnt2	0.38	0.006	0.05	Pdgfrb	-0.23	0.050	0.40				
88									Kcna1	-0.38	0.010	0.07	Dnmt3a	0.22	0.034	0.38				
89									Nkd1	-0.38	0.004	0.04	Npffr2	0.22	0.044	0.40				
90									Col23a1	0.38	0.005	0.04	St18	-0.22	0.050	0.40				
91									Clu	0.38	0.001	0.01	Dvl2	0.21	0.032	0.38				
92									Nxph4	0.37	0.008	0.06	Mid1	0.20	0.046	0.40				
93									Htr1a	0.37	0.004	0.04	Myt1l	0.19	0.042	0.40				
94									Cadps2	0.37	0.004	0.04								
95									Rprm	0.37	0.010	0.07								
96									Baiap3	0.37	0.007	0.06								
97									Col5a2	0.37	0.005	0.04								
98									Kcnq3	-0.37	0.006	0.05								
99									Vat1	0.36	0.008	0.06								

100									Cacna1h	0.36	0.013	0.08								
101									Hcn1	0.36	0.012	0.08								
102									Sema6a	-0.36	0.008	0.06								
103									Lgi3	-0.36	0.016	0.10								
104									Ndnf	0.36	0.011	0.08								
105									Plagl1	0.36	0.006	0.05								
106									Kctd12	0.36	0.013	0.08								
107									Sh3bgrl2	-0.36	0.016	0.10								
108									Gad1	0.36	0.009	0.07								
109									Rorb	-0.36	0.011	0.08								
110									Cerk	-0.35	0.007	0.06								
111									Cdh13	0.35	0.011	0.08								
112									Chst2	-0.35	0.008	0.06								
113									Epha4	-0.35	0.013	0.08								
114									Htr2c	0.35	0.014	0.09								
115									Ctss	0.35	0.011	0.08								
116									Cux2	-0.35	0.008	0.06								
117									Ngfr	0.34	0.009	0.07								
118									Sema3a	0.34	0.023	0.13								
119									Zbtb20	0.34	0.015	0.09								
120									Slit2	0.34	0.012	0.08								
121									Lmo3	0.34	0.024	0.13								
122									Mc4r	0.34	0.015	0.09								
123									Pex5l	-0.34	0.025	0.13								
124									Ngef	0.34	0.015	0.09								
125									Gpr56	0.33	0.017	0.10								
126									Map2	0.33	0.011	0.08								
127									Pcdh20	0.33	0.016	0.10								
128									Sncg	0.33	0.022	0.12								
129									Nrn1	-0.32	0.012	0.08								
130									Pcdh18	0.32	0.015	0.09								
131									Pvalb	-0.32	0.020	0.11								
132									Lypd1	0.32	0.028	0.15								
133									Grik5	0.32	0.043	0.19								
134									Zic1	-0.32	0.043	0.19								
135									Unc5d	0.31	0.017	0.10								
136									Snca	0.31	0.026	0.14								
137									Vegfa	-0.31	0.020	0.11								
138									Slc18a2	0.31	0.039	0.18								
139									Btrc	0.31	0.039	0.18								
140									Tshz1	-0.31	0.022	0.12								
141									Gpc3	0.31	0.024	0.13								
142									Zbtb7b	-0.31	0.040	0.18								
143									Pou6f1	0.31	0.028	0.15								
144									Tacr1	0.31	0.020	0.11								
145									Ednrb	0.30	0.023	0.13								
146									Cds1	-0.30	0.032	0.16								
147									Adcy2	0.30	0.030	0.15								
148									Dkk3	0.30	0.041	0.19								
149									B3galt2	-0.30	0.039	0.18								
150									Pdgfra	0.30	0.034	0.17								
151									Fibcd1	0.30	0.044	0.19								
152									Grin3a	0.30	0.037	0.18								
153									Gpr123	-0.29	0.035	0.17								
154									Nfasc	-0.29	0.034	0.17								
155									Lrrn3	0.29	0.036	0.18								
156									Tead1	0.29	0.019	0.11								
157									Gfra1	0.29	0.018	0.11								
158									Ascl1	0.29	0.041	0.19								
159									Nhlh2	-0.29	0.013	0.08								
160									Col11a1	0.29	0.039	0.18								
161									Rcn1	0.29	0.039	0.18								
162									Ntrk1	0.29	0.043	0.19								
163									Ptprz1	0.29	0.045	0.19								
164									Pole4	0.29	0.044	0.19								
165									Gpx1	-0.29	0.028	0.15								
166									Pde10a	-0.28	0.030	0.15								
167									Foxm1	-0.28	0.024	0.13								
168									Crtac1	0.28	0.045	0.19								
169									Wnt5a	0.27	0.048	0.21								
170									Plp1	-0.26	0.020	0.11								
171									Sema6d	-0.26	0.040	0.18								
172									Rest	0.26	0.042	0.19								
173									Trp73	-0.26	0.033	0.17								
174									Vav2	0.25	0.034	0.17								
175									Sorl1	0.25	0.038	0.18								
176									Lhx3	-0.25	0.036	0.18								
177									Zbtb7a	-0.25	0.043	0.19								

Table S1. Genes with high similarity to source wiring PI gradients.

The table lists genes with high cosine similarity to each of the source wiring PI components ($p < 0.05$). Q-values from false discovery rate (FDR) correction are also provided. For space limitations, only genes within the top 50 ranks are shown. See Supporting Information Dataset 1 for details.

	PI target 1				PI target 2				PI target 3				PI taergt 4				PI target 5			
Rank	Gene	Sim	*p*	*q*	Gene	Sim	*p*	*q*	Gene	Sim	*p*	*q*	Gene	Sim	*p*	*q*	Gene	Sim	*p*	*q*
1	Ly6h	0.82	0.001	0.03	Efnb2	-0.72	0.001	0.04	Gpr56	0.70	0.001	0.04	Igfbp5	-0.43	0.010	1.00	Col1a1	0.53	0.001	0.38
2	Grm5	0.81	0.001	0.03	Cnr1	-0.71	0.001	0.04	Nlk	-0.69	0.001	0.04	Tacr1	0.43	0.004	1.00	Vat1	-0.51	0.001	0.38
3	Grik5	0.81	0.001	0.03	Cadps2	-0.71	0.001	0.04	Ece2	0.67	0.001	0.04	Nrp1	-0.40	0.010	1.00	Nr4a3	0.50	0.002	0.51
4	Cpne7	0.80	0.001	0.03	Sema3a	-0.67	0.001	0.04	Baiap3	0.66	0.001	0.04	Pde10a	0.40	0.015	1.00	Sncg	-0.50	0.004	0.51
5	Grin2b	0.78	0.001	0.03	Sema6a	0.67	0.001	0.04	Prkcb	-0.66	0.001	0.04	Pbx3	0.39	0.025	1.00	Zfpm2	0.50	0.003	0.51
6	Camk2a	0.78	0.002	0.04	Amotl1	0.67	0.001	0.04	Dlk1	0.65	0.001	0.04	Col15a1	-0.38	0.030	1.00	Cdon	0.48	0.004	0.51
7	Pcdh19	0.78	0.001	0.03	Tcf7l2	0.66	0.001	0.04	Cds1	-0.64	0.003	0.06	Astn2	-0.37	0.037	1.00	Clcn5	-0.48	0.013	0.94
8	Cacna2d1	0.77	0.001	0.03	Reln	-0.64	0.001	0.04	Zbtb7a	-0.64	0.001	0.04	Cd9	-0.37	0.018	1.00	Adnp2	0.46	0.019	0.94
9	Dlg4	0.76	0.001	0.03	Slc17a6	0.64	0.002	0.05	Etv5	-0.62	0.001	0.04	Stx3	-0.37	0.033	1.00	Slc16a2	0.45	0.011	0.93
10	Lhx1	-0.75	0.002	0.04	Tcf4	-0.63	0.002	0.05	Slc6a11	0.62	0.001	0.04	Mylk	-0.37	0.017	1.00	Slc18a2	-0.44	0.028	0.94
11	Rprm	0.74	0.001	0.03	Inha	-0.63	0.001	0.04	Dusp14	-0.62	0.003	0.06	Tshz1	0.36	0.021	1.00	Bmpr1a	0.44	0.010	0.93
12	Astn1	0.74	0.001	0.03	Satb2	-0.61	0.003	0.06	Myt1	0.61	0.002	0.05	Prox1	-0.36	0.038	1.00	Etv1	0.43	0.040	0.94
13	Pdgfra	0.74	0.001	0.03	Zfhx3	0.61	0.001	0.04	Fbxw7	-0.61	0.001	0.04	Aldh2	-0.36	0.019	1.00	Kctd12	0.43	0.036	0.94
14	Nrgn	0.74	0.001	0.03	Grik3	-0.60	0.001	0.04	Grin2a	-0.61	0.001	0.04	Itih3	-0.35	0.046	1.00	Bcl9	0.43	0.040	0.94
15	Egr1	0.73	0.001	0.03	Id2	-0.60	0.002	0.05	Pik3r1	-0.60	0.002	0.05	Tead3	-0.34	0.031	1.00	Abat	-0.43	0.020	0.94
16	Top1	0.73	0.001	0.03	Pdgfb	-0.59	0.001	0.04	Kif5c	-0.60	0.002	0.05	Eya4	-0.32	0.007	1.00	Id3	0.42	0.006	0.65
17	Gria1	0.73	0.005	0.08	Plekhg1	0.59	0.001	0.04	Ddit4l	-0.60	0.002	0.05	Gja1	-0.31	0.050	1.00	Ache	-0.42	0.041	0.94
18	Ctss	0.73	0.001	0.03	Kit	-0.59	0.001	0.04	Kcnn2	-0.60	0.001	0.04	Atf5	-0.31	0.033	1.00	Mef2c	0.42	0.033	0.94
19	Hopx	-0.72	0.002	0.04	Bmp3	-0.59	0.006	0.08	Lrp1	-0.60	0.001	0.04	C1qb	-0.27	0.050	1.00	Fbxw4	0.41	0.037	0.94
20	Pcdh1	0.72	0.004	0.07	Kctd12	-0.59	0.003	0.06	Satb2	-0.60	0.003	0.06					Slc17a7	0.40	0.048	0.94
21	Gria2	0.71	0.001	0.03	Fezf2	-0.58	0.004	0.07	Mxi1	-0.60	0.003	0.06					Nfil3	0.40	0.037	0.94
22	Gabrb3	0.70	0.001	0.03	Mafb	-0.58	0.005	0.07	Plxnd1	-0.60	0.009	0.09					Abca2	-0.39	0.036	0.94
23	Apc	0.70	0.001	0.03	S1pr1	-0.58	0.002	0.05	Gtf2h5	-0.59	0.008	0.09					Gabra5	0.39	0.046	0.94
24	Syn1	0.70	0.002	0.04	Nfix	-0.58	0.004	0.07	Efnb3	0.59	0.001	0.04					Ddc	-0.39	0.037	0.94
25	Scg5	0.70	0.001	0.03	Tyro3	-0.57	0.009	0.09	Ccng1	-0.59	0.001	0.04					Pomgnt1	0.38	0.036	0.94
26	Cdh11	0.70	0.001	0.03	Ngef	-0.57	0.004	0.07	Calb2	0.58	0.001	0.04					Chrm2	-0.37	0.045	0.94
27	Actb	0.70	0.001	0.03	Pvrl1	-0.57	0.001	0.04	Gsg1l	-0.58	0.002	0.05					Cd164	-0.37	0.047	0.94
28	Cacna1h	0.69	0.003	0.05	Pde5a	-0.57	0.003	0.06	Jdp2	-0.58	0.004	0.07					Ppfibp1	0.36	0.039	0.94
29	Cdh13	0.69	0.001	0.03	Lef1	0.56	0.001	0.04	Prss12	-0.58	0.003	0.06					Reln	0.36	0.049	0.94
30	Cdh8	0.69	0.001	0.03	Neurod1	-0.56	0.002	0.05	Pou6f1	-0.58	0.003	0.06					Gjd2	-0.36	0.016	0.94
31	Kcnk2	0.69	0.003	0.05	Hcn1	-0.56	0.002	0.05	Snw1	-0.58	0.005	0.07					Fmo1	0.33	0.039	0.94
32	Vgf	0.69	0.001	0.03	Parm1	-0.56	0.001	0.04	Pak1	-0.57	0.002	0.05					Dvl2	0.30	0.031	0.94
33	Snca	0.69	0.003	0.05	Zfhx4	0.56	0.001	0.04	Satb1	-0.57	0.002	0.05								
34	Zfp521	-0.69	0.002	0.04	Scg3	-0.56	0.001	0.04	Id2	-0.57	0.006	0.08								
35	Kctd9	-0.69	0.003	0.05	Lxn	-0.55	0.001	0.04	Mafb	-0.57	0.004	0.07								
36	Ahi1	0.69	0.003	0.05	Ntng1	0.55	0.003	0.06	B3galt2	-0.57	0.002	0.05								
37	Mapt	0.68	0.002	0.04	Tbr1	-0.55	0.005	0.07	Cux2	-0.57	0.002	0.05								
38	Cyc1	0.68	0.006	0.09	Lrrc4	-0.55	0.003	0.06	Large	-0.56	0.008	0.09								
39	App	0.68	0.002	0.04	Rab3b	-0.54	0.002	0.05	Egr2	-0.56	0.004	0.07								
40	Chrm3	0.68	0.004	0.07	Wnt7b	-0.54	0.003	0.06	Tbr1	-0.56	0.008	0.09								
41	Maged1	0.67	0.002	0.04	Mybpc1	-0.54	0.003	0.06	Sorl1	-0.56	0.005	0.07								
42	Nrg1	-0.67	0.002	0.04	Arc	-0.54	0.008	0.09	Zbtb16	-0.56	0.005	0.07								
43	Egr3	0.67	0.003	0.05	Pole4	-0.53	0.005	0.07	Gsk3a	-0.56	0.001	0.04								
44	Slit1	0.66	0.004	0.07	Qdpr	0.53	0.003	0.06	Dusp6	-0.56	0.003	0.06								
45	Pcsk2	0.66	0.002	0.04	Nlk	-0.53	0.003	0.06	Nfix	-0.56	0.005	0.07								
46	Nkx2-3	0.66	0.034	0.30	Inpp4b	0.53	0.008	0.09	Arc	-0.55	0.009	0.09								
47	Btrc	0.65	0.009	0.12	Mpped1	-0.52	0.015	0.13	Glcci1	-0.55	0.005	0.07								
48	Nucb2	0.65	0.007	0.10	Thrb	-0.52	0.008	0.09	Igfbp4	-0.55	0.011	0.10								
49	Grid1	0.64	0.002	0.04	Itgb8	-0.52	0.004	0.07	Scube1	-0.55	0.011	0.10								
50	Atp8a1	0.64	0.010	0.13	Car10	-0.52	0.002	0.05	Mapk8	-0.55	0.007	0.09								
51	Tox3	0.64	0.002	0.04	Ppp1r1b	-0.52	0.005	0.07	Kcna1	-0.54	0.007	0.09								
52	Mycbp2	0.64	0.012	0.16	Mterf2	-0.52	0.005	0.07	Ctnnd1	-0.54	0.005	0.07								
53	Zdhhc2	0.63	0.005	0.08	Nefh	0.51	0.009	0.09	Rhoa	-0.54	0.002	0.05								
54	Pou3f2	0.63	0.008	0.12	Pou6f1	-0.51	0.005	0.07	Nkx2-3	-0.54	0.014	0.11								
55	Glra1	-0.63	0.039	0.33	Whrn	-0.51	0.003	0.06	Robo2	-0.53	0.010	0.10								
56	Mapk8ip3	0.62	0.013	0.16	Ets2	-0.51	0.008	0.09	Bcor	-0.53	0.002	0.05								
57	Psd	0.62	0.009	0.12	Ptprz1	-0.51	0.007	0.09	Col5a1	-0.53	0.010	0.10								
58	Pcdhga12	0.62	0.009	0.12	B3gnt2	-0.51	0.010	0.10	Setd7	-0.53	0.005	0.07								
59	Aplp1	0.62	0.023	0.24	Trp53i11	-0.51	0.003	0.06	Ephb6	-0.53	0.024	0.14								
60	Dusp6	0.62	0.040	0.33	Lzts1	-0.50	0.013	0.12	Sparc	0.53	0.013	0.11								
61	Rgs4	0.62	0.011	0.14	Col5a1	-0.50	0.009	0.09	Slc1a2	-0.53	0.008	0.09								
62	Ephb6	0.61	0.027	0.26	Zfp516	-0.50	0.013	0.12	Plxna2	-0.53	0.019	0.13								
63	Csnk1a1	0.61	0.014	0.17	Slc6a1	-0.50	0.007	0.09	Lrrc4	-0.53	0.017	0.13								
64	Celsr3	0.61	0.004	0.07	Scube1	-0.50	0.010	0.10	Chrm3	-0.52	0.019	0.13								
65	Alcam	0.61	0.022	0.24	Ptprk	-0.50	0.009	0.09	Ntm	-0.52	0.006	0.08								
66	Pknox2	0.61	0.026	0.25	Lgi3	0.50	0.020	0.15	Tle4	-0.52	0.003	0.06								
67	Onecut3	0.60	0.030	0.28	Cnp	0.50	0.014	0.13	Coch	-0.52	0.005	0.07								
68	Clstn1	0.60	0.018	0.20	Slc9a9	-0.50	0.007	0.09	Sel1l	-0.52	0.001	0.04								
69	Nos3	0.60	0.023	0.24	Chrm1	-0.50	0.009	0.09	Rspo1	-0.52	0.006	0.08								
70	Chrm4	0.60	0.013	0.16	Chl1	-0.50	0.004	0.07	Nckap1	-0.52	0.007	0.09								
71	Spp1	-0.60	0.020	0.22	Jazf1	-0.49	0.012	0.11	Pcdhb9	-0.52	0.006	0.08								
72	Nde1	0.60	0.043	0.34	Pknox2	-0.48	0.013	0.12	Pex5l	-0.52	0.014	0.11								
73	Adcy2	0.60	0.030	0.28	Bcl11a	-0.48	0.024	0.15	Epha4	-0.52	0.008	0.09								
74	Lrp6	0.60	0.008	0.12	Sp3	-0.48	0.018	0.14	Csnk1a1	-0.52	0.006	0.08								
75	Asic2	0.60	0.014	0.17	Plcg1	-0.48	0.010	0.10	Foxp1	-0.52	0.009	0.09								
76	Kcnq3	0.59	0.018	0.20	Sowaha	-0.48	0.015	0.13	Rptor	-0.52	0.004	0.07								
77	Slc1a2	0.59	0.021	0.23	Nfil3	-0.48	0.011	0.11	Nrn1	-0.52	0.027	0.14								
78	Cers4	0.59	0.024	0.24	Slc12a2	0.47	0.017	0.14	Arntl	-0.51	0.009	0.09								
79	Lmo3	0.59	0.032	0.29	Ubtf	-0.47	0.023	0.15	Mpped1	-0.51	0.029	0.15								
80	Npy	0.59	0.043	0.34	Dscaml1	-0.47	0.022	0.15	Pknox2	-0.51	0.010	0.10								
81	Ets2	0.59	0.034	0.30	Lrp8	-0.47	0.006	0.08	Ecel1	0.51	0.021	0.13								
82	Satb1	0.58	0.035	0.30	Fibcd1	-0.47	0.023	0.15	Lifr	-0.51	0.013	0.11								
83	Ctnnb1	0.58	0.024	0.24	Vip	-0.47	0.020	0.15	Bcl11a	-0.51	0.029	0.15								
84	Unc5d	0.58	0.022	0.24	S100a10	-0.47	0.023	0.15	Mef2c	-0.51	0.014	0.11								
85	Loxl2	-0.58	0.041	0.33	Rspo1	-0.47	0.006	0.08	Kcnq3	-0.51	0.006	0.08								
86	Ssrp1	0.57	0.024	0.24	Foxk2	-0.47	0.021	0.15	Tiam2	-0.51	0.012	0.11								
87	Erbb4	0.57	0.025	0.25	Gria2	-0.46	0.024	0.15	Mlx	-0.50	0.020	0.13								
88	Col4a5	-0.56	0.005	0.08	Ddit4l	-0.46	0.022	0.15	Dkk3	-0.50	0.021	0.13								
89	Lypd1	0.56	0.035	0.30	Thra	-0.46	0.017	0.14	Rapgef5	-0.50	0.023	0.13								
90	Epha4	0.56	0.044	0.34	Slc6a11	0.46	0.022	0.15	Mib2	-0.50	0.004	0.07								
91	Nrxn1	0.55	0.043	0.34	Car2	0.46	0.016	0.13	Htr2c	0.50	0.021	0.13								
92	Col4a1	0.55	0.049	0.36	Pthlh	-0.46	0.023	0.15	Gria3	-0.50	0.023	0.13								
93	Aebp2	0.55	0.017	0.20	Gfra2	-0.46	0.005	0.07	Itih3	0.50	0.017	0.13								
94	Cdh2	0.54	0.039	0.33	Gdf10	-0.46	0.023	0.15	Fgfr1	0.50	0.004	0.07								
95	Zfp207	0.54	0.048	0.36	Dkk3	-0.45	0.027	0.16	Mybpc1	-0.50	0.024	0.14								
96	Unc5b	-0.54	0.030	0.28	Ache	0.45	0.032	0.17	Mef2d	-0.50	0.038	0.17								
97	Bhlhe40	0.54	0.040	0.33	Cyr61	-0.45	0.020	0.15	Clstn1	-0.50	0.012	0.11								
98	Hmg20a	0.54	0.044	0.34	Large	-0.45	0.024	0.15	Sema4g	0.50	0.033	0.16								
99	Esrrg	-0.53	0.045	0.34	Cpne2	-0.45	0.008	0.09	Ppp1r1b	-0.50	0.027	0.14								
100	Pcdh10	0.53	0.044	0.34	L1cam	0.45	0.015	0.13	Clstn2	-0.50	0.011	0.10								
101	Rnf14	0.51	0.032	0.29	Map2	-0.45	0.012	0.11	Nts	0.50	0.017	0.13								
102	Elf1	0.50	0.015	0.18	Psd	-0.45	0.010	0.10	Hoxd3	-0.49	0.021	0.13								

103	Cdh24	0.50	0.050	0.37	Ptk7	-0.44	0.022	0.15	Slitrk1	-0.49	0.013	0.11
104	Tcf25	0.48	0.034	0.30	Ednrb	-0.44	0.016	0.13	Slc17a7	-0.49	0.009	0.09
105					Col23a1	-0.44	0.027	0.16	Nnat	0.49	0.017	0.13
106					Kcnk2	-0.44	0.034	0.17	Srebf2	-0.49	0.008	0.09
107					Met	-0.44	0.029	0.16	Dbi	0.49	0.013	0.11
108					Mef2d	-0.44	0.028	0.16	Zeb2	-0.49	0.014	0.11
109					Hhex	-0.44	0.011	0.11	Bhlhe40	-0.49	0.022	0.13
110					Asic1	-0.43	0.016	0.13	Chrm1	-0.49	0.037	0.17
111					Etv5	-0.43	0.042	0.19	Zbtb20	0.48	0.009	0.09
112					Foxf2	-0.43	0.030	0.16	Sstr2	-0.48	0.026	0.14
113					Ntrk3	-0.43	0.030	0.16	Pomgnt1	-0.48	0.010	0.10
114					Egr3	-0.43	0.037	0.18	Fezf2	-0.48	0.035	0.16
115					Nos3	-0.43	0.028	0.16	Nos1ap	-0.48	0.010	0.10
116					Bmp4	-0.43	0.022	0.15	Ikzf5	-0.48	0.023	0.13
117					Nr4a3	-0.43	0.021	0.15	Tcf4	-0.48	0.024	0.14
118					Sertad2	-0.43	0.006	0.08	Fam3c	-0.48	0.050	0.20
119					Ctss	-0.43	0.027	0.16	mCG146432	-0.48	0.022	0.13
120					Zfp445	-0.43	0.017	0.14	Ptprk	-0.48	0.032	0.15
121					Gabrg2	-0.43	0.026	0.16	Gpx3	0.48	0.031	0.15
122					Phf13	-0.43	0.006	0.08	Foxk2	-0.48	0.026	0.14
123					Crym	-0.42	0.049	0.21	Asic1	-0.48	0.019	0.13
124					Boc	-0.42	0.018	0.14	Kcnj6	-0.48	0.026	0.14
125					Grm2	-0.42	0.033	0.17	Tyro3	-0.48	0.036	0.16
126					Max	-0.42	0.020	0.15	Adnp2	-0.48	0.016	0.12
127					Sorl1	-0.42	0.009	0.09	Gal	0.48	0.045	0.18
128					Etv1	-0.42	0.038	0.18	Egr3	-0.47	0.031	0.15
129					Ece2	0.42	0.037	0.18	Cdh11	-0.47	0.021	0.13
130					Cit	0.42	0.024	0.15	Cebpd	-0.47	0.025	0.14
131					Glcci1	-0.42	0.022	0.15	Itpr1	-0.47	0.019	0.13
132					Mga	-0.42	0.021	0.15	Slc9a9	-0.47	0.042	0.17
133					Kcnc2	0.42	0.028	0.16	Sox8	-0.46	0.038	0.17
134					Pcdhga12	-0.42	0.043	0.19	Crebbp	-0.46	0.011	0.10
135					Egr1	-0.42	0.030	0.16	Ntng2	-0.46	0.021	0.13
136					Tpd52l1	0.42	0.023	0.15	Mgst3	-0.46	0.027	0.14
137					Atf5	-0.42	0.004	0.07	Fat3	-0.46	0.034	0.16
138					Nr1i2	-0.41	0.033	0.17	Tfdp2	-0.46	0.028	0.14
139					Tal1	0.41	0.025	0.15	Thrb	-0.46	0.043	0.18
140					Fgfrl1	-0.41	0.042	0.19	Gpc3	0.46	0.022	0.13
141					Prkcd	0.41	0.045	0.20	Grm4	-0.46	0.028	0.14
142					Kmt2c	-0.41	0.030	0.16	Plcg1	-0.46	0.031	0.15
143					Ctgf	-0.41	0.036	0.18	Creb3	-0.45	0.005	0.07
144					Atp8a1	-0.41	0.038	0.18	Ank3	-0.45	0.009	0.09
145					Slc16a2	-0.41	0.024	0.15	Cttnbp2	-0.45	0.050	0.20
146					Onecut2	-0.41	0.043	0.19	Peg10	0.45	0.023	0.13
147					Btbd11	0.40	0.032	0.17	Gfra2	-0.45	0.016	0.12
148					Cntn3	-0.40	0.046	0.20	Cadm3	-0.45	0.031	0.15
149					Glul	0.40	0.043	0.19	Cux1	-0.45	0.022	0.13
150					Lamp5	-0.40	0.046	0.20	Rgs4	-0.45	0.032	0.15
151					Eomes	-0.40	0.023	0.15	Nek7	-0.45	0.016	0.12
152					Rasa2	-0.40	0.027	0.16	Dvl1	-0.45	0.024	0.14
153					Sel1l	-0.40	0.007	0.09	Cck	-0.45	0.019	0.13
154					Nos1	-0.40	0.045	0.20	Eya4	-0.45	0.003	0.06
155					Chrm2	0.40	0.027	0.16	Egr1	-0.44	0.028	0.14
156					Tle4	-0.40	0.034	0.17	Zfp46	-0.44	0.048	0.19
157					Zdhhc2	-0.40	0.040	0.19	Sncg	0.44	0.020	0.13
158					Gria1	-0.40	0.045	0.20	Csnk1d	-0.44	0.050	0.20
159					Pik3r1	-0.40	0.030	0.16	Laptm4b	-0.44	0.036	0.16
160					Lrrn3	-0.40	0.031	0.17	Cdh7	-0.44	0.029	0.15
161					Slc17a7	-0.40	0.040	0.19	Maz	-0.44	0.012	0.11
162					Gad1	-0.39	0.029	0.16	Nr2c2	-0.43	0.023	0.13
163					En2	-0.39	0.039	0.19	Plcxd2	-0.43	0.040	0.17
164					Rprm	-0.38	0.050	0.21	Chrna5	-0.43	0.046	0.19
165					Aldh2	-0.38	0.021	0.15	Arnt2	-0.43	0.033	0.16
166					Evx1	-0.38	0.027	0.16	Zkscan6	-0.43	0.040	0.17
167					Polr3k	-0.38	0.033	0.17	Sertad2	-0.43	0.021	0.13
168					Dcun1d5	-0.38	0.001	0.04	Rorb	-0.43	0.036	0.16
169					Fstl1	0.38	0.046	0.20	Top1	-0.43	0.040	0.17
170					Arnt2	-0.38	0.041	0.19	Lrp6	-0.43	0.039	0.17
171					Dlx1	-0.38	0.041	0.19	Nsd1	-0.43	0.025	0.14
172					Unc5d	-0.37	0.039	0.19	Pvrl3	-0.43	0.037	0.17
173					Nsd1	-0.37	0.033	0.17	Vegfa	-0.42	0.028	0.14
174					Pcdh20	-0.37	0.043	0.19	Spint2	0.42	0.048	0.19
175					Npffr2	-0.37	0.035	0.18	Mta2	-0.42	0.035	0.16
176					Fam189a2	0.36	0.050	0.21	Boc	-0.42	0.041	0.17
177					Trp53	-0.35	0.038	0.18	Epha10	-0.41	0.040	0.17
178					Plp1	0.35	0.025	0.15	Abl1	-0.41	0.038	0.17
179					Nrg2	-0.35	0.049	0.21	Cntn1	-0.41	0.038	0.17
180					Gatad1	-0.35	0.049	0.21	Dvl3	-0.41	0.018	0.13
181									Klf6	-0.41	0.040	0.17
182									Pvalb	-0.41	0.046	0.19
183									Tcf25	-0.41	0.019	0.13
184									Jag1	-0.40	0.018	0.13
185									Dvl2	-0.40	0.023	0.13
186									Mid1	-0.40	0.026	0.14
187									Arl10	0.40	0.040	0.17
188									Gabpb1	-0.40	0.031	0.15
189									Asic2	-0.39	0.046	0.19
190									Gja1	0.38	0.041	0.17
191									Whsc1	-0.38	0.032	0.15
192									Myt1l	-0.37	0.019	0.13
193									Dnmt3a	-0.35	0.024	0.14
194									Dcun1d5	-0.31	0.026	0.14

Table S2. Genes with high similarity to target wiring PI gradients.

The table lists genes with high cosine similarity to each of the target wiring PI components ($p < 0.05$). Q-values from false discovery rate (FDR) correction are also provided. For space limitations, only genes within the top 50 ranks are shown. See Supporting Information Dataset 1 for details.

Gene set	Short link of query URL
All 763 genes	https://biit.cs.ut.ee/gplink/l/aeMZrqglyQc
PI source 1	https://biit.cs.ut.ee/gplink/l/aVk7kkG2gRj
PI source 2	https://biit.cs.ut.ee/gplink/l/aLOnSNgw5SS
PI source 3	https://biit.cs.ut.ee/gplink/l/aEB0Zz9I1Se
PI source 4	https://biit.cs.ut.ee/gplink/l/a9YICRs-4Qb
PI source 5	https://biit.cs.ut.ee/gplink/l/aH6Lr0WOtQ8
PI target 1	https://biit.cs.ut.ee/gplink/l/aS49ESgqPRn
PI target 2	https://biit.cs.ut.ee/gplink/l/aYAQ-AaxwQV
PI target 3	https://biit.cs.ut.ee/gplink/l/a7Ujs7_cjSp
PI target 4	https://biit.cs.ut.ee/gplink/l/aCe3OCw3IQq
PI target 5	https://biit.cs.ut.ee/gplink/l/azz5otoxMRG
All PI	https://biit.cs.ut.ee/gplink/l/a1mSqgRhdQ1

Table S3. Query links of gene ontology (GO) enrichment analysis.

The table lists short URLs corresponding to the query links used in GO enrichment analyses conducted with g:Profiler. Detailed analysis settings and results can be accessed via these URLs.
